# Analysis and Potential Value of Compounds Extracted From Star Ruby, Rio Red, and Ruby Red Grapefruit, and Grapefruit Juice Processing Residues *via* Steam Explosion

**DOI:** 10.3389/fnut.2021.691663

**Published:** 2021-09-13

**Authors:** Christina Dorado, Randall G. Cameron, John A. Manthey, Jinhe Bai, Kyle L. Ferguson

**Affiliations:** U.S. Horticultural Research Laboratory, United States Department of Agriculture, Agricultural Research Service, Fort Pierce, FL, United States

**Keywords:** valorization, pectin, limonene, extraction, flavonoid, *Citrus paradisi*

## Abstract

Culled whole grapefruit (WG) and grapefruit juice processing residues (GP) are currently incorporated into low-cost animal feed. If individual chemical components found within these side streams could be recovered as high-value coproducts, this would improve the overall value of the grapefruit crop. In this study, pectic hydrocolloids, sugars, volatiles, phenolics, and flavonoids were extracted from Star Ruby, Rio Red, and Ruby Red GP and WG using a continuous pilot scale steam explosion system. Up to 97% of grapefruit juice oils and peel oils could be volatilized and contained 87–94% *d*-limonene. The recovery of pectin, as determined by galacturonic acid content, was between 2.06 and 2.72 g 100 g^−1^. Of the phenolics and flavonoids analyzed in this study, narirutin and naringin were extracted in the amounts of up to 10,000 and 67,000 μg g^−1^, respectively.

## Introduction

Florida, Texas, and California are the major producers of grapefruit (*Citrus paradisi* Macfad.) in the United States, where approximately 256,000 metric tons were collectively cultivated during the 2018–19 season (1). Red grapefruit varieties dominate, and up to 61% of the cultivated grapefruit was processed (1–3). Grapefruit is typically processed into juice but can also be converted into segments and salads (1, 4–6). When grapefruit is processed into juice, 51% of the fruit is left behind in the form of rind (peel), rag, seeds, and membranes (7). Currently, citrus juice processors convert these residues to low-value animal feed (8) through the most energy-intensive operation of the juice-processing plant (9). The result is little to no-profit margin when energy costs are high. However, grapefruit process residues contain valuable pectic hydrocolloids, sugars, volatiles, phenolics, and flavonoids that are lost when the residues are converted to animal feed. If these compounds could be extracted from the residues, these high-value chemicals could bring new revenue streams to grapefruit processing plants and improve profit margins. To accomplish this, steam explosion was explored as a means of extracting maximum amounts of these high-value compounds. Pectic hydrocolloids, sugars, volatiles, phenolics, and flavonoids have been extracted from orange juice-processing residues previously, using a continuous pilot scale steam explosion system (10–12). This previous study has focused only on the sweet orange varieties used for orange juice processing in Florida. Sweet orange fruit and grapefruit not only differ in color and size but also in the quality and quantity of specific components associated with their composition. Therefore, it would be expected that the pectic hydrocolloids, sugars, volatiles, phenolics, and flavonoids that can be extracted using steam explosion of grapefruit will differ from sweet orange fruit. Considering that the majority of grapefruit cultivated in the U.S. is of the red varieties, the application of steam explosion for the extraction of value-added compounds from whole fruit and juice-processing remnants of red grapefruit was deemed appropriate. In this study, we describe the use of a continuous pilot scale steam explosion system as a proof-of-concept study for processing of Star Ruby, Rio Red, and Ruby Red grapefruit from California, Texas, and Florida for the extraction of pectic hydrocolloids, sugars, volatiles, phenolics, and flavonoids.

## Materials and Methods

### Star Ruby, Rio Red, and Ruby Red Grapefruit

Packing house fruit was prepared for fresh market sale by treatment with fungicides and wax to improve shelf life and appearance and was used as received. This allowed for simulation of steam explosion of packing house fruit that does not meet the stringent standards for fresh market sale and could ultimately be processed for value-added compound extraction. Star Ruby grapefruit from a packing house in Tulare County, California, was acquired for these experiments. The grapefruit was treated with either thiabendazole, imazalil, fludioxonil, or azoxystrobin and coated with food-grade beeswax or vegetable-based wax. Rio Red grapefruit from a packing house in Mission, Texas, was also acquired for these experiments. The grapefruit was coated with a food-grade vegetable and or lac-resin-based wax or resin. Thiabendazole and/or orthophenylphenate were used as fungicides. Ruby Red whole grapefruit and Ruby Red grapefruit juice-processing residues were acquired from a Florida juice-processing plant for these experiments. Fruit that was split open or falling apart was culled, and the remaining fruit was rinsed with water and scrubbed gently with a scouring pad to remove dirt. Images of the grapefruit used in these experiments can be found in Supplementary Figures 1–3. The average mass of the whole fruit, peel, and juice can be found in Supplementary Table 1. A sample of cold-pressed and centrifuged oil from Rio Red grapefruit peel from a juice processor in Mission, Texas, was acquired and analyzed for density and composition as described below and reported for comparison.

### Steam Explosion of Star Ruby, Rio Red, and Ruby Red Grapefruit Juice-Processing Residues (GP) and Whole Grapefruit (WG)

Star Ruby or Rio Red grapefruit was cut in half and juiced by hand, using a Sunkist Juice Extractor (Model# 8-RA07 or 8-RC00, Ontario, CA, USA). Ruby Red-juiced grapefruit halves were acquired from a Florida juice processing plant and used as they were received. The juiced grapefruit halves, from here on described as grapefruit juice-processing residues or GP, were put into five-gallon buckets. Star Ruby, Rio Red, and Ruby Red grapefruit were cut into quarters or eighths, from here on described as whole grapefruit or WG, and were placed in five-gallon buckets. Buckets of WG or GP were closed with a bucket lid and stored at room temperature or a walk-in refrigerator (6°C) for no more than 3 h. The GP or WG was loaded into the hopper of a continuous pilot scale steam explosion system. GP or WG was added to the hopper based on the flow rate of the material. The material flow rate is not controlled but is dependent on the steam injection and the release of material at the pneumatic valve. If the material fed into the hopper is hard and thick, the flow is impaired, but if the material is soft and thin, the flow rate is improved. This is most likely due to decomposition of the material in the presence of heat and steam during the process. The use of this continuous steam explosion process was described previously (10). Briefly, GP or WG was subjected to saturated steam at a temperature of 140–145°C and pumped through a hold tube to a pneumatic back-pressure relief valve with a set point of 50 psi. The back-pressure relief valve is made up of a pneumatic rack and pinion actuator (Model# PAVCL253S-0115, PBM, Inc., Irwin, PA, USA) and a flow control positioner (Model# APEX 8000A81137AOT, Flowserve, Corp., Cookeville, TN, USA). The hold tube is 15.24 m long, with a diameter of 7.26 cm, resulting in hold times of approximately 1–3 min. Upon release of pressure, GP or WG was vented into a flash tank and pumped into resealable gallon bags and stored at −24°C. Any volatiles released upon flashing of GP or WG were condensed and collected in a separate tank. An image of front and top views of the continuous pilot scale steam explosion system can be found in Supplementary Figure 4. The parameters for steam explosion of GP or WG of each variety can be found in Supplementary Table 2.

### Soluble and Compositional Sugar Analysis

Fresh or steam-treated GP and WG samples (25–50 g) were diluted to 25% (w/v) in deionized water. Diluted samples were prepared for sugar analysis by size reduction in a Waring Commercial Blender (Model# 7011S).

Compositional sugars (glucose, fructose, and galacturonic acid) were determined by hydrolysis of 10 g of the diluted sample in 1.25 ml of 50-mmol L^−1^ sodium acetate buffer, pH 4.8, and 1.25 ml of deionized water, using 100 μl each of two different pectinases (DSM, PAC, Batch 16B04V1, pectinase activity, 49.43 U ml^−1^, and Rapidase PNS, pectinase activity, 58.29 U ml^−1^), 50 μl cellulase (Novozyme, Cellic CTec2, VCPI0003, cellulase activity, 208.21 FPU ml^−1^), and 50 μl β-glucosidase (Novozyme 188, DCN00205, β-glucosidase activity, 270.67 U ml^−1^) enzymes with rotation for 24 h at 45°C. To prevent microbial growth, 37 μl of cycloheximide (5 mg ml^−1^ stock) and 37 μl of chloroamphenicol (10 mg ml^−1^ stock) were added. Samples were then filtered, using a 0.45 μm GD/X Nylon syringe filter to remove insoluble solids prior to analysis.

The water extract of diluted samples was prepared for soluble sugars (glucose, fructose, and sucrose) analysis by first removing insoluble solids by filtration, using a 0.45 μm GD/X Nylon syringe filter.

Soluble and compositional sugars were quantified and identified by direct high-performance ion exchange chromatography (HPIEC) analysis of the clarified extracts. Samples were analyzed by pulsed-amperometric detection (PAD) on an Antec Decade Elite system, with the thermal chamber set to 30°C and configured to accept two analytical columns attached to separate electrochemical cells (AntecFlexcel Au HyRef) in a parallel configuration without post-column eluent (13). Two high-performance liquid chromatography (HPLC) systems PAD 1 and PAD 2 were configured for PAD on the Antec Decade Elite system. PAD 1 consisted of a quaternary pump (Agilent 1260 QuatPump VL), temperature-controlled auto sampler set to 4°C (Agilent Infinity ALS auto sampler with Infinity Thermostat), and a column compartment set to 25°C (Agilent Infinity 1290 TCC). PAD 2 consisted of a quaternary pump (Agilent Infinity 1260 QuatPump VL), an auto sampler with temperature control set to 4°C (Agilent Infinity II 1260 VialSampler), and a column compartment set to 25°C (Agilent Infinity II 1260 MCT). Samples were injected into both PAD 1 and PAD 2 at a volume of 5 μl. The HPLC method for both PAD 1 and PAD 2 utilized three buffers. Buffer A consisted of 18 mM sodium hydroxide, buffer B consisted of 100 mM sodium hydroxide/150 mM sodium acetate, and buffer C consisted of 200 mM sodium hydroxide. The percent of each buffer used, time, and flow for the sugar analysis can be seen in Supplementary Table 3. The waveform method was the same for both electrochemical cells and can be seen in Supplementary Table 4. The waveform method and temperature settings were input manually, using the Antec Decade Elite system digital display. Data collection and analysis were completed, using Agilent OpenLab CDS Chemstation Rev C. The method was calibrated at the beginning of each sequence run. Three levels of standard (0.01, 0.02, and 0.03%) were prepared from a stock solution of rhamnose, arabinose, galactose, glucose, xylose, fructose, sucrose, cellobiose, and galacturonic acid. An internal standard of 2-deoxy-D-galactose (0.01%) was used to compensate for system-wide changes in response during the sequence run. The calibration curve was calculated by the Agilent Chemstation software, using a linear regression forced through zero. Correlation coefficients of 0.995 or greater were achieved. Chromatograms of standards run at the beginning of each series of runs and a standard run at the end of each series of runs were overlapped to ensure that elution time and peak shapes were consistent through a series of runs. All standards were purchased from Sigma-Aldrich. The samples were analyzed in triplicate. A sample chromatogram can be seen in Supplementary Figure 5.

### Dry Weight Determination

Total dry weight was determined gravimetrically by oven-drying at 70°C for a minimum of 24 h, followed by 1 h at 70–75°C under a vacuum on single samples.

### Juice Oil and Peel Oil Concentration

The Scott oil test is based on a bromination reaction to determine the number of fatty acid bonds present in a sample and can give a good estimate of oil content when *d*-limonene content is high (9). The concentration of oil in fresh or steam-treated GP and WG was determined, using the Scott bromate titration method with some modifications (14). Briefly, a standard *d*-limonene solution was prepared, using 100 μl *d*-limonene in 100 ml of isopropanol. Then, 10 ml of the standard *d*-limonene solution was diluted in 25 ml of deionized water and 15 ml of isopropanol. The standard solution was then refluxed for approximately 30 min, and the alcohol portion was collected. To the alcohol portion, 10 ml of 50% HCl and two to three drops of methyl orange indicator were added. This was then titrated with 0.0247 N bromate-bromide solution to a colorless endpoint. The factor was calculated using Equation 1:


(1) $$\begin{array}{r} \frac{0.01\;ml\;of\;d-limonene\;in\;10\;ml\;of\;standard\;solution}{ml\;of\;titrant\;used}=factor \end{array}$$

In order to minimize the use of titrant used, fresh or steam-treated GP and WG samples were diluted. A weighed sample (25–50 g) was first diluted in deionized water to 25% (w/v). The diluted sample was mixed in a Waring Commercial Blender (Model# 7011S) until a smooth puree was achieved. Then, 21 ml of deionized water was added to 4 g of the puree. This was then further diluted with 25 ml of deionized water and 25 ml of isopropanol for a total of 75 ml volume.

The 75 ml prepared solution was then refluxed for approximately 30 min, and the alcohol portion was collected. To the alcohol portion, 10 ml of 50% HCl and two to three drops of methyl orange indicator were added. This was then titrated with 0.0247 N bromate-bromide solution to a colorless endpoint.

The amount of *d*-limonene was calculated using Equation 2:


(2) $$\begin{array}{r} \left(\frac{\left\{factor\;from\;Equation\;1\;x\;ml\;of\;titrant\;used\right\}}{\;g\;of\;sample}\right)X\;100=\%\frac{v}{w} \end{array}$$

Averages of triplicate analysis and SDs were reported.

### Condensed Volatile Density and Composition

The condensed vapors from the steam explosion system were collected in a separate tank and allowed to cool and separate overnight. The following day, a valve at the bottom of the separation tank was opened, and the lower aqueous layer was drained as much as possible before collecting the upper organic layer. The organic and aqueous layers were allowed to further separate in a separatory funnel with occasional mixing to break up any emulsions that may have formed. The density of the separated organic layer was determined by measuring the mass of a 2 or 5 ml Gay–Lussac bottle (Pyrex) filled with the organic layer. Triplicate measurements and the corresponding SD are reported. The volatile composition analysis of the top organic layer was carried out, using gas chromatography–mass spectrometry (GC-MS, 6890N GC and 5975 MS; Agilent, Santa Clara, CA, USA), equipped with a split/splitless injector and a DB-5 column (60 m × 0.25 mm i.d., film thickness = 1 μm; J&W Scientific, Folsom, CA, USA). The injection volume was 1 μl with a split ratio of 40:1, and the injector temperature was 250°C. The column oven temperature was programmed from 40°C (0.5 min) to 250°C (13.25 min) at a rate of 4°C min^−1^, then ramped at 100°C min^−1^ to 260°C, and held for 4 min for a total run time of 63 min. Helium was used as the carrier gas at a flow rate of 1.5 ml min^−1^. For MS settings, inlet, ionizing source, and transfer line temperatures were kept 250, 230, and 280°C, respectively. MS data were recorded in the scan mode from 40 to 400 m/z at 2 scans s^−1^ with an ionization energy of 70 eV (15). Data were collected using the ChemStation G1701 AA data system (Hewlett-Packard, Palo Alto, CA, USA). A mixture of C-5 to C-18 *n*-alkanes was run at the beginning of each day to calculate retention indices (RIs) (15). The volatile components were identified by matching their spectra with those from the National Institute of Standards and Technology (NIST)/Environmental Protection Agency (EPA)/National Institutes of Health (NIH) Mass Spectral Library (NIST 14; WebBook, SRD69) and authentic volatile compound standards, as well as by comparing their RIs with corresponding literature data (16). Quantification of limonene was conducted by using a peak size (total ion currents) vs. a concentration curve built by a series of diluted standard solutions, ranging from 1.6 to 2.0% (17), and the samples were diluted to 2% by methanol. All other compound contents were calculated based on the ratio of total ion currents. Average peak area percent based on total ion current of at least triplicate analyses was reported for selected compounds. The peak area percent of each compound for each replicate can be found in Supplementary Tables 5–8. A sample chromatogram can be seen in Supplementary Figure 6.

### Pectin Extraction and Analysis

#### Acid Extraction of Pectin From Fresh Peel

Fresh grapefruit peel was cut into ~1–3 cm pieces. In a 1 L jacketed reaction vessel, fresh peel was added to water heated to 70°C. A peel/water ratio of 1:4.3 was used for pectin extraction. Concentrated nitric acid was used to adjust the pH to 1.8. The slurry was stirred for 3 h, adjusted to pH 2.2 with 5 M NaOH, and then filtered through two layers of cheesecloth. The pectin in the filtrate was precipitated with two volumes of propan-2-ol overnight at room temperature. The precipitate was centrifuged at 15,000 × g for 20 min at 20°C. The pectin pellets were frozen at −20°C and then lyophilized (FreeZone Freeze Dry System; Labconco, Kansas City, MO, USA).

#### Recovery of Pectic Hydrocolloids

Pectic hydrocolloids were recovered from the steam-exploded biomass, using a simple water wash (10, 18). Three replicates of equal weights of steam-exploded biomass and water (100 g each) were mixed and placed on a wrist shaker for 30 min at room temperature. Chloramphenicol (10 mg ml^−1^) and cycloheximide (5 mg ml^−1^) were added to prevent microbial growth. The slurries were centrifuged at a relative centrifugal force of 15,000 × g for 20 min at 4°C. The supernatant was recovered, and the pellet was washed two more times as described above, with 100-g deionized water, for a total of three washes per replicate. An aliquot of each wash supernatant was used for determination of its carbohydrate composition *via* enzymatic hydrolysis, in duplicate (12). The remaining portions from each wash were pooled and preserved with lithium azide (0.02%, wt/wt). Residual solids were removed by filtration through 1.2 μm glass filter fiber (GF/C, Whatman/GE Healthcare Life Sciences, Ltd., Great Britain). Pectic hydrocolloids contained within the pooled supernatants were recovered by precipitation with acidified ethanol (55% final concentration) overnight at 4°C (19). The precipitated pectic hydrocolloids were centrifuged at 15,000 × g for 20 min at 20°C. The pellets were washed in acidified ethanol and centrifuged as described above. The washed pellets were lyophilized and then stored at −80°C.

#### Macromolecular Characterization of Pectic Hydrocolloids

Size exclusion chromatography, using a model 1260 Infinity autosampler and pump (Agilent Technologies, Germany), coupled to a HELEOS II Multiangle laser light-scattering photometer (MALLS; Wyatt Technology, Santa Barbara, CA, USA), a Viscostar II differential pressure viscometer (DP; used to estimate intrinsic viscosity), and an Optilab differential refractive index detector (dRI; Wyatt Technology, Santa Barbara, CA, USA), was used to determine number average (M_n_) and weight average (M_w_) molecular weight and intrinsic viscosity [η] (10). A minimum of three replicates were run, and the data were statistically analyzed by two-way ANOVA and means separated where appropriate using Tukey's multiple comparison (GraphPad Prizm, version 4.00 for Windows, GraphPad Software, San Diego, CA, USA, www.graphpad.com).

#### Determination of Degree of Methylesterification (DM)

The degree of methylesterification (DM) was determined using a modified method outlined in Anthon and Barrett (20) and Quesenberry and Lee (21). Briefly, a buffered copper solution, containing 23.2 g NaCl, 2.3 g sodium acetate, and 1 ml glacial acetic acid, was made in 80 ml of deionized water, then adjusted to pH 4.8 with 3 M NaOH, and brought to 100 ml. The buffered copper solution (50 μl), a volume of a 0.1% w/v digested pectin solution (6 μl), and a sodium acetate buffer (44 μl) were added to each well in a 96-well microplate (Corning Inc., Corning, NY, USA) and mixed. The microplate was sealed with an adhesive seal and heated for 90 min at 90°C. Afterward, the microplate was cooled to room temperature. Then, 200 μl of a 5.0 mM bicinchoninic acid (BCA) solution in 250 mM, a pH 10.1 carbonate buffer, was added to each well. Galacturonic acid absorbance was measured at 550 nm (20).

Methanol concentration was determined, using alcohol oxidase and Purpald (22) with modifications. Digested pectin solution (0.1% w/v, 6 μl) and of 200 mM, a pH 7 phosphate buffer (84 μl) were added to microplate wells. Then, a 2 U ml^−1^ of alcohol oxidase (10 μl; Sigma-Aldrich, St. Louis, MO, A2404-1KU) solution was added to each well. To allow for full conversion of methanol to formaldehyde, the microplate was incubated for 30 min at room temperature (21). Then, a 34 mM Purpald solution (100 μl) was mixed into each well. To determine the formaldehyde concentration in each well, the color was allowed to develop in the dark at room temperature for 20 min. The reaction was quenched by the addition of 33 mM NaIO_4_ and then read at 550 nm (20). Galacturonic acid and methanol calibration curves (5, 10, 20, and 25 μM, three replicates each) were included with each microplate. A positive pectin control, with a known DM, was also included (Sigma-Aldrich, P9315, Lot # 75H1111). The DM of pectin was calculated using Equation 3.


(3) $$\begin{array}{r} DM\;=\;\frac{\left(Concentration\;of\;methanol\right)}{\left(Concentration\;of\;galacturnoic\;acid\right)}*100 \end{array}$$

### Phenolic and Flavonoid Analysis

High-performance liquid chromatography-photodiode array mass spectrometry (HPLC-PDA-MS) analysis of methanol and water extracts of fresh and steam-exploded Star Ruby, Rio Red, and Ruby Red GP and WG were completed. Water extracts were prepared by taking a 1:3 ratio of deionized water to fresh and steam-exploded Star Ruby, Rio Red, and Ruby Red GP and WG and homogenizing and or blending (Omni International homogenizer, Model GLH-01, Omni International, Marietta, GA, USA or Waring Commercial Blender, Model# 7011S, Waring Commercial, Tarrington, CT, USA). The samples were microcentrifuged, and the supernatants were used for analysis. Methanol extracts were prepared by taking 1.5 g of fresh Star Ruby, Rio Red, and Ruby Red GP or WG and homogenizing (Omni International homogenizer, Model GLH-01, Omni International, Marietta, GA, USA) in approximately 30 ml of methanol. The sample was then vacuum filtered. This was repeated two times more, and the filtered extracts were pooled and brought to 100-ml volume with methanol. A 15 ml portion was dried in a concentrator (SpeedVacConcentrator, SVC 200H, Thermo Scientific, Waltham, MA, USA) and brought to 4 ml with dimethylsulfoxide and was used for analysis. HPLC-PDA-MS analysis of methanol and water extracts of fresh and steam-exploded Star Ruby, Rio Red, and Ruby Red GP and WG was completed as described previously (18). The flavanones naringin-4′-O-glucoside, hesperidin glucoside, narirutin, naringin, naringin-6”-malonate, isosakuranetinrutinoside, and poncirin were quantified and qualified by UV-Vis at a wavelength of 285 nm. The coumarins dihydroxy-osthol and marmin were quantified and qualified by UV-Vis at a wavelength of 320 nm. Averages of triplicate flavonoid analyses and their SDs were reported. The values used to calculate the averages and SDs can be found in Supplementary Tables 9–13 of the Supporting Information section. A sample chromatogram can be seen in Supplementary Figure 7.

## Results and Discussion

### Soluble and Compositional Sugars

Steam explosion of Star Ruby, Rio Red, and Ruby Red WG increased the amount of glucose and fructose as a result of the breakdown of sucrose when compared with fresh WG (Supplementary Figures 8A,B). There was little to no effect on the yield of the soluble sugars for all three varieties of GP subjected to steam explosion as compared to water extraction of fresh GP (Supplementary Figures 8C,D). The combination of steam explosion and enzymes did increase the yield of glucose by 33% and fructose by 28% for Rio Red GP (Supplementary Figure 8D) as compared to enzyme alone (Supplementary Figure 9C) but had little to no effect on the yield of glucose and fructose for the Star Ruby and Ruby Red varieties.

#### Potential Value of Sugars

While steam explosion did not yield a marked increase in the amount of sugars extracted from WG or GP as compared with water extraction of fresh WG or GP, steam explosion did allow for the simultaneous extraction and isolation of other valuable compounds, such as pectin, volatiles, phenolics, and flavonoids. The extracted sugars can be converted to commodity chemicals rather than discarded. This will address any costs or environmental issues associated with the disposal of the sugars and will result in an increase in the profitability of processing the residues. For example, if glucose from steam-exploded GP is converted to ethanol at 50% yield (23), then that would equate to a value of approximately 68,000 USD for Star Ruby GP, 272,000 USD for Rio Red GP, and 192,000 USD for Ruby Red GP based on the 2018–2019 citrus processing season (1) and the 2019 wholesale price of ethanol (24). These cumulative values were based on processed red grapefruit from FL and total processed grapefruit from TX and CA, since the National Agricultural Statistics Service (NASS) does not distinguish between red and white grapefruits for those two states (1).

### Juice Oil, Peel Oil, and Volatiles

#### Juice Oil and Peel Oil Concentration

**Figure 1:**
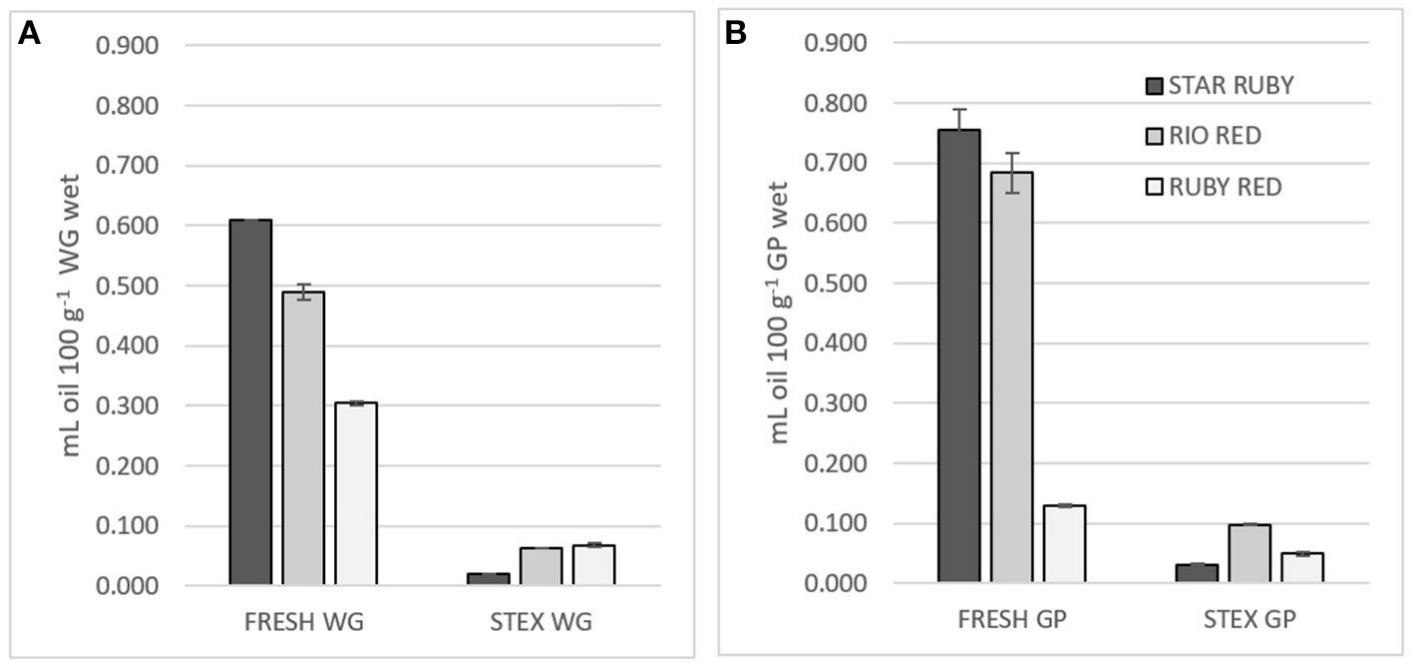
Juice oil and peel oil content of fresh (FRESH) and steam exploded (STEX) Star Ruby, Rio Red and Ruby Red **(A)** whole grapefruit (WG) and **(B)** grapefruit juice processing residues (GP). Absence of error bars indicates the Standard Error of the Mean was too small to be visible.

As the name suggests, peel oil is found in the peel of citrus fruits, particularly in sacs within the flavedo (6). There are also small amounts of oil (0.008 and 0.075%) found in the juice (25) that differ in composition to that found in the peel (26). Oil content followed the trend Star Ruby > Rio Red >> Ruby Red in fresh WG and GP (Figure 1). Ruby Red GP had much less oil than the Star Ruby and Rio Red varieties. The extraction method can also impact the amount of oil found in the juice-processing residues (26). The Star Ruby and Rio Red varieties were allocated from packing houses and juiced by hand, whereas the Ruby Red GP was obtained from a juice-processing plant. The significantly lower oil content of Ruby Red GP was most likely due to the de-oiling procedures that take place at the juice-processing plant and the use of industrial equipment for juicing (8). Steam explosion not only leads to fragmentation of the grapefruit feedstock but also to volatilization of the oil. Steam explosion volatized 95% of the oil in Star Ruby WG and GP, 84% of the oil in Rio Red WG and GP, and 56–75% of the oil in Ruby Red WG and GP.

#### Condensed Volatiles Composition and Density

The peel oil, juice oil, and other volatiles that exist in WG and GP are volatilized as a result of the steam explosion process and are then condensed and collected in a separate tank. The composition of the condensed volatiles is reported in Table 1. The composition of grapefruit oils and volatiles can vary depending on the variety, source location, and stage of maturity (27–30). The major constituent in the condensed volatiles of grapefruit peel is *d*-limonene and is present in amounts ranging from 60 to 95% (31). In our study, *d*-limonene ranged from 87.06 to 93.73% with lower values of *d*-limonene present in the Star Ruby and Ruby Red GP samples (Table 1). In the literature, the amount of *d*-limonene in red grapefruit varieties can range from 76.09 to 96.06% for cold pressed and distilled peel (27, 29, 32). Interestingly, when lower values of *d*-limonene were observed, increased amounts of β-myrcene and decanal were found in our samples as well as in the literature. The volatile profiles of the GP and WG for Ruby Red and Rio Red varieties were very similar. This was not the case with Star Ruby variety with increased amounts of nonanal, decanal, carvone, geranyl acetate, α-copaene, β-elemene, (*E*)-caryophyllene, and δ-cadinene in GP as compared with WG. Even though three different varieties of red grapefruit from three different states were used in this study, the densities of the condensed volatiles were very similar and only ranged from 0.8431 to 0.8477 g ml^−1^ (Table 2). These densities are very close to the density of *d*-limonene (33) but were lower than what has been reported for cold pressing, hydrodistillation, and solvent-free microwave extraction of grapefruit peel, which ranged from 0.853 to 0.883 g ml^−1^ (Table 2). It may be that the cold pressed and hydrodistilled samples in particular exhibit greater densities due to the presence of water or other more dense compounds. For example, Ahmed reported densities of 0.853–0.862 g ml^−1^ for hydrodistilled oil but had low *d*-limonene concentrations of 76.09–78.73% (27). Grapefruit oil is generally found to consist of >95% *d*-limonene (9), but steam-exploded and hydrodistilled grapefruit samples had <95% *d*-limonene (Table 1). The *d*-limonene may have degraded from exposure to extended periods of high temperature and high pressure. Steam distillation at high temperatures and pressures can lead to degradation of *d*-limonene, the major chemical constituent in peel oil, to alcohols and epoxides (9). In fact, when we look at the total oxygenated compounds (Table 1), we see that significantly greater amounts of these compounds were produced in the higher temperature steam explosion experiments of the Star Ruby GP and WG (Table 1).

**Table 1:** Composition of condensed volatiles from the steam explosion of Star Ruby, Rio Red and Ruby Red whole grapefruit (WG) and grapefruit juice processing residues (GP).

**Reference**							**28**		**26**				**31**
Process	Steam Explosion						Cold Pressed	Distilled	Hydrodistillation			Cold Pressed Centrifuged	Cold Pressed
Variety	Ruby Red		Rio Red		Star Ruby		Star Ruby	Star Ruby	Star Ruby	Ruby Red	Rio Red	Rio Red	Redblush
Location	Florida		Texas		California		Taiwan	Taiwan	India			Texas	Kenya
Feedstock	GP	WG	GP	WG	GP	WG	Dry Flavedo	Dry Peel	Dry Peel			GP	Flavedo
**Compound**	**% based on total ion current**												
α-Pinene	0.36	0.49	0.72	0.58	0.40	0.58	0.85	0.52	1.73	1.44	1.01	0.62	0.5
β-Myrcene^a^	1.66	1.70	2.40	2.17	1.38	1.74	3.06	2.06	6.24	5.61	4	1.79	nr
Octanal	0.07	0.21	0.22	0.24	0.04	0.08	nr	nr	0.93	1.01	0.71	0.28	0.3
D-Limonene	87.94	91.43	91.24	91.31	87.06	93.73	91.83	96.06	78.73	76.09	nd	93.17	91.1
Linalool	0.16	0.18	0.20	0.17	0.14	0.12	nd	nd	0.31	0.31	0.17	0.09	0.1
Nonanal	0.07	0.03	0.03	0.04	0.09	0.05	nr	nr	0.23	0.26	0.16	0.08	0.1
Decanal	0.07	0.04	0.16	0.03	1.38	0.83	nd	0.39	1.44	1.45	0.85	nd	0.3
Carvone	1.60	0.52	0.17	0.13	0.11	0.02	nd	nd	nr	nr	nr	nd	0.1
Geranyl acetate	0.12	0.10	0.14	0.08	0.48	0.11	nr	nr	0.36	nd	0.09	0.05	nr
α-Copaene	0.32	0.22	0.17	0.15	0.65	0.18	0.2	nd	0.41	nd	0.24	0.16	nr
β-Elemene	0.28	0.12	0.12	0.12	0.50	0.10	nd	nd	nr	nr	nr	0.16	nr
(E)-Caryophyllene^b^	0.89	0.71	0.36	0.46	1.52	0.57	nr	nr	1.06	nd	0.45	0.40	nr
δ-Cadinene^c^	0.27	0.22	0.12	0.18	0.75	0.20	nr	0.28	0.44	nd	0.26	0.18	nr
Total	93.81	95.97	96.05	95.66	94.50	98.31	95.94	99.31	91.88	86.17	7.94	96.98	92.50
Oxygenated Total^d^	2.09	1.08	0.92	0.69	2.24	1.21	0.00	0.39	3.27	3.03	1.98	0.50	0.90

a *Compound Identified as β-Myrcene or Myrcene*.

b *Compound Identified as (E)-Caryophyllene or Carophyllene*.

c *Compound Identified as δ-Cadiene or β-Cadiene*.

d *Total of Octanal, Linalool, Nonanal, Decanal, Carvone, Geranyl Acetate*.

*nr, not reported*.

*nd, not detected*.

**Table 2:** Density of condensed volatiles from the steam explosion of Star Ruby, Rio Red and Ruby Red whole grapefruit (WG) and grapefruit juice processing residues (GP).

**Reference**								**29**		**33**	**26**		
Process	Steam Explosion						Cold Press/Centrifuged	HD	SFME	HD	HD		
Variety	Ruby Red		Rio Red		Star Ruby		Rio Red	nr	nr	nr	Ruby Red	Rio Red	Star Ruby
Location	Florida		Texas		California		Texas	Turkey	Turkey	Nigeria	India		
Feedstock	WG	GP	WG	GP	WG	GP	GP	Peel	Peel	Rind	Dry Peel		
Density (g mL^−1^)	0.8444	0.8477	0.8431	0.8437	0.8438	0.8473	0.8527	0.856	0.862	0.883	0.854	0.861	0.862
Standard Error (±)	0.0004	0.0002	0.0006	0.0002	0.0004	0.0009	0.0001	nr	nr	nr	0.20	0.15	0.49

*HD, Hydrodistillation*.

*SFME, Solvent Free Microwave Extraction*.

*nr, not reported*.

#### Potential Value of Oil and Condensed Volatiles

Citrus juice-processing plants can extract oil at various stages of the processing line (8). While oil is present in small amounts relative to the mass of the grapefruit as a whole, it commands a high-dollar value, making it a worthwhile commodity to collect. For example, at the end of the 2019 season, pink grapefruit oil was selling for 75 USD/kg (34). We have demonstrated that steam explosion is capable of volatilizing 56–95% of the oil in WG and GP and that the volatiles collected exhibit similar amounts of *d*-limonene to that of cold pressed and hydrodistilled grapefruit peel oils (Table 1). Based on the amount of oil that can be volatilized and theoretically collected, using the continuous pilot-scale steam explosion system, the potential value that could have been obtained for Star Ruby GP for the 2018–2019 season would have equated to approximately 4.6 million USD. For Rio Red GP, the potential value would have equated to 22 million USD, and for Ruby Red GP, the potential value would have equated to 1.7 million USD. These cumulative values were based on the processed red grapefruit from FL and the total processed grapefruit from TX and CA, since NASS does not distinguish between red and white grapefruits for those two states (1).

### Pectin

#### Recovery of Pectic Hydrocolloids From Steam Explosion

**Figure 2:**
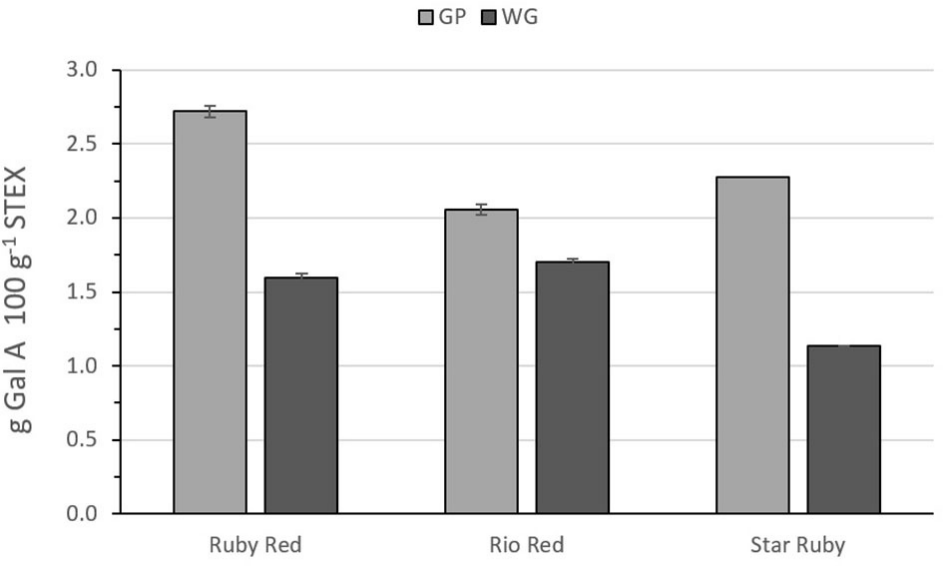
Recovery of pectic hydrocolloids from steam exploded whole grapefruit (WG) and grapefruit juice processing residues (GP) as determined by the amount of galacturonic acid in wash supernatants after normalizing for % dry wt. Absence of error bars indicates the Standard Error of the Mean was too small to be visible.

Figure 2 shows the recovery of pectic hydrocolloids, following steam explosion of either GP or WG between variety and biomass type. In all cases, normalizing the recoveries on a percentage dry weight basis, more pectic hydrocolloids were recovered from GP alone vs. WG. Differences between recoveries from GP vs. WG were most pronounced for Ruby Red and Star Ruby. Recoveries from WG were relatively similar but were lower in Star Ruby. GP from Ruby Red yielded the highest recovery.

#### Macromolecular Properties of Pectic Hydrocolloids

**Figure 3:**
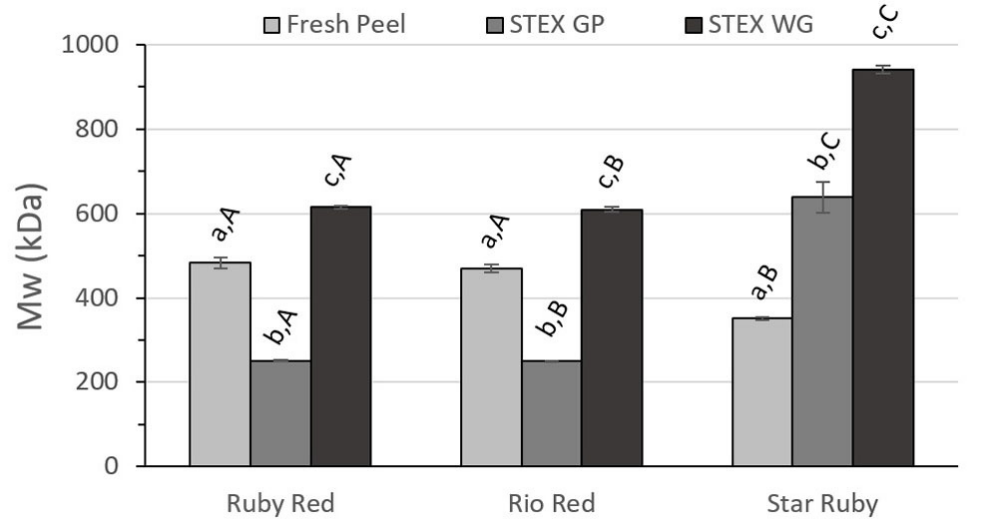
Weight average molecular weight (M_w_) of recovered pectic hydrocolloids from acid extracted fresh peel, and steam exploded (STEX) whole grapefruit (WG) and grapefruit juice processing residues (GP). Absence of error bars indicates the Standard Error of the Mean was too small to be visible. Bars with different lower case letters indicate a statistically significant difference (*p* > 0.05) within fruit from each individual variety, bars with different upper case letters indicate a statistically significant difference (*p* > 0.05) for different varieties for each type of extraction.

**Figure 4:**
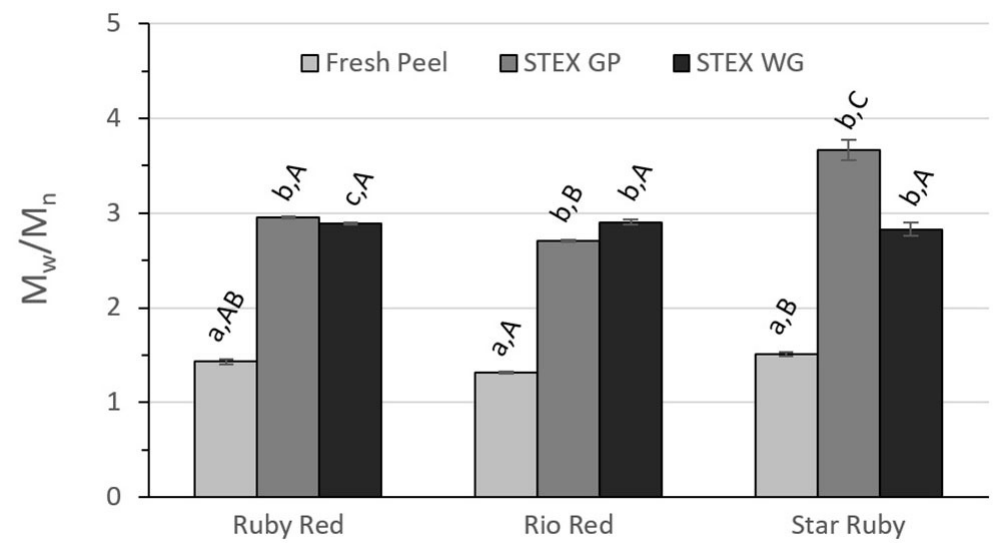
Polydispersity index (M_w_/M_n_) of recovered pectic hydrocolloids from acid extracted fresh peel, and steam exploded (STEX) whole grapefruit (WG) and grapefruit juice processing residues (GP). Absence of error bars indicates the Standard Error of the Mean was too small to be visible. Bars with different lower case letters indicate a statistically significant difference (*p* > 0.05) within fruit from each individual variety, bars with different upper case letters indicate a statistically significant difference (*p* > 0.05) for different varieties for each type of extraction.

**Figure 5:**
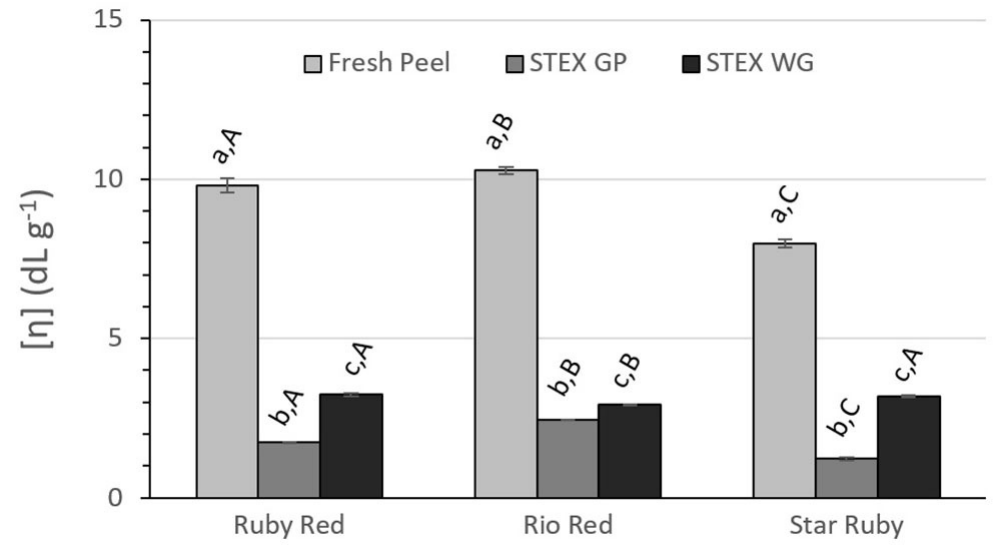
Intrinsic viscosity [η] of recovered pectic hydrocolloids from acid extracted fresh peel, and steam exploded (STEX) whole grapefruit (WG) and grapefruit juice processing residues (GP). Absence of error bars indicates the Standard Error of the Mean was too small to be visible. Bars with different lower case letters indicate a statistically significant difference (*p* > 0.05) within fruit from each individual variety, bars with different upper case letters indicate a statistically significant difference (*p* > 0.05) for different varieties for each type of extraction.

Pectin functionality is dependent on its M_w_, polydispersity index (M_w_/M_n_), [η], and DM. Polydispersity index (M_w_/M_n_) and [η] for acid extracted and steam exploded pectic hydrocolloids were analyzed by two-way ANOVA (Figures 3, 4, and 5). For all of these variables, the analyses indicated statistically significant effects due to variety (*p* < 0.0001), the extraction method (*p* < 0.0001), and the interaction between the variety of the grapefruit and the extraction method (*p* < 0.0001). Tukey's multiple comparison test indicated significant differences for grapefruit from the different varieties and for steam explosion of GP vs. WG vs. acid extraction from fresh peel.

Differences in M_w_ between the extraction method and biomass type (fresh peel, steam exploded GP and steam exploded WG) were similar for Ruby Red and Rio Red (Figure 3). In both of these groups, there were significant differences for all biomass types and for steam exploded biomass type between varieties. Results from Star Ruby differed from Ruby Red and Rio Red in that M_w_ of pectic hydrocolloids from acid extracted raw peel was lower than the other varieties (*p* > 0.05) and that M_w_ from steam exploded GP (*p* > 0.05) and WG (*p* > 0.001) was higher than the other varieties.

The polydispersity of the recovered pectic hydrocolloids from steam exploded biomass was significantly greater than from acid extracted and fresh peel pectin from every variety (Figure 4). The polydispersity of Rio Red and Star Ruby fresh peel differed (*p* > 0.05), but neither was significantly different from Ruby Red fresh peel. A significant difference for polydispersity index was only observed between steam exploded GP vs. steam exploded WG from Ruby Red (*p* > 0.05). No significant differences were observed for polydispersity index for any variety for pectic hydrocolloids recovered from steam explosion of WG, but all varieties were significantly different from each other for steam exploded GP (*p* > 0.05).

Intrinsic viscosity [η] decreased significantly for pectic hydrocolloids isolated from steam exploded biomass vs. acid extraction from fresh peel (Figure 5) in all varieties (*p* > 0.001). Significant differences were observed between steam exploded GP and WG within each variety (*p* > 0.001 for Ruby Red and Rio Red, *p* > 0.01 for Star Ruby). Between varieties, all were significantly different for steam exploded GP (*p* > 0.0001 for Ruby Red vs. Rio Red; *p* > 0.05 for Ruby Red vs. Star Ruby; *p* > 0.01 for Rio Red vs. Star Ruby) and Rio Red was different from both Ruby Red and Star Ruby for steam exploded WG (*p* > 0.05).

All pectic material extracted from this red grapefruit biomass had DM values >50% (Supplementary Figure 9). Within a variety, there were a few statistically significant differences for DM among the treatments. For all the varieties, the acid extracted, fresh peel pectin had a lower DM than the pectic hydrocolloids from steam explosion of WG (*p* > 0.05). The DM of acid extracted pectin from Star Ruby fresh peel was also significantly different from pectic hydrocolloids from steam explosion of GP (*p* > 0.05). Pectic hydrocolloids from the two different steam explosion treatments were not significantly different for any variety. Also, there were no significant differences between varieties for any extraction method.

The three macromolecular properties reported to be responsible for pectin functionality are molecular weight, degree of methylesterification, and the distribution of charge within the homogalacturonan region (35, 36). Although M_w_ of pectic hydrocolloids did increase following steam explosion of WG, the M_w_ from steam explosion of GP was lower than either acid extracted pectin from fresh peel or steam exploded WG, except for Star Ruby where steam exploded GP had an M_w_ higher than pectin from fresh peel. The increase in polydispersity and decrease in [η], following steam explosion, suggest these pectic hydrocolloids could have potential applications beyond traditional use of pectin in food formulation (37–40).

#### Potential Value of Grapefruit Pectic Hydrocolloids Obtained by Steam Explosion

Compared with pectin from lemon or lime fruit peel, grapefruit pectin tends to have a lower [η] value, but it also possesses a higher calcium sensitivity, which is useful for applications containing acidified dairy proteins (41, 42). These calcium sensitive pectins can be valued at 19–22 USD per kg (43). The pectic hydrocolloids from steam explosion of GP or WG may also be useful for pharmaceutical, nutraceutical, or personal care products. Other potential applications include ion binding (44, 45) or hydration control (36, 46). The recovery of galacturonic acid following steam explosion was 2.7199 g 100 g^−1^ from Ruby Red GP, 2.0567 g 100 g^−1^ from Rio Red GP, and 2.2777 g 100 g^−1^ from Star Ruby GP (Figure 2). Values for steam explosion of WG were not estimated since processed fruits are used for juice production, and GP is the coproduct. Based on a ratio of dry weight of the fresh peel to dry weight of the steam-exploded GP and production numbers for the 2018–2019 harvest season (1), we can estimate a recovery of approximately 230, 256, and 47 metric tons, respectively, of Ruby Red, Rio Red, and Star Ruby GP. Assigning a value of 20 USD per kg for calcium sensitive pectin, this suggests a potential maximal value of approximately 4.6, 5.1, and 0.94 million USD, respectively, for Ruby Red, Rio Red, and Star Ruby GP. These cumulative values were based on processed red grapefruit from FL and total processed grapefruit from TX and CA, since NASS does not distinguish between red and white grapefruits for those two states (1).

### Phenolics and Flavonoids

**Figure 6:**
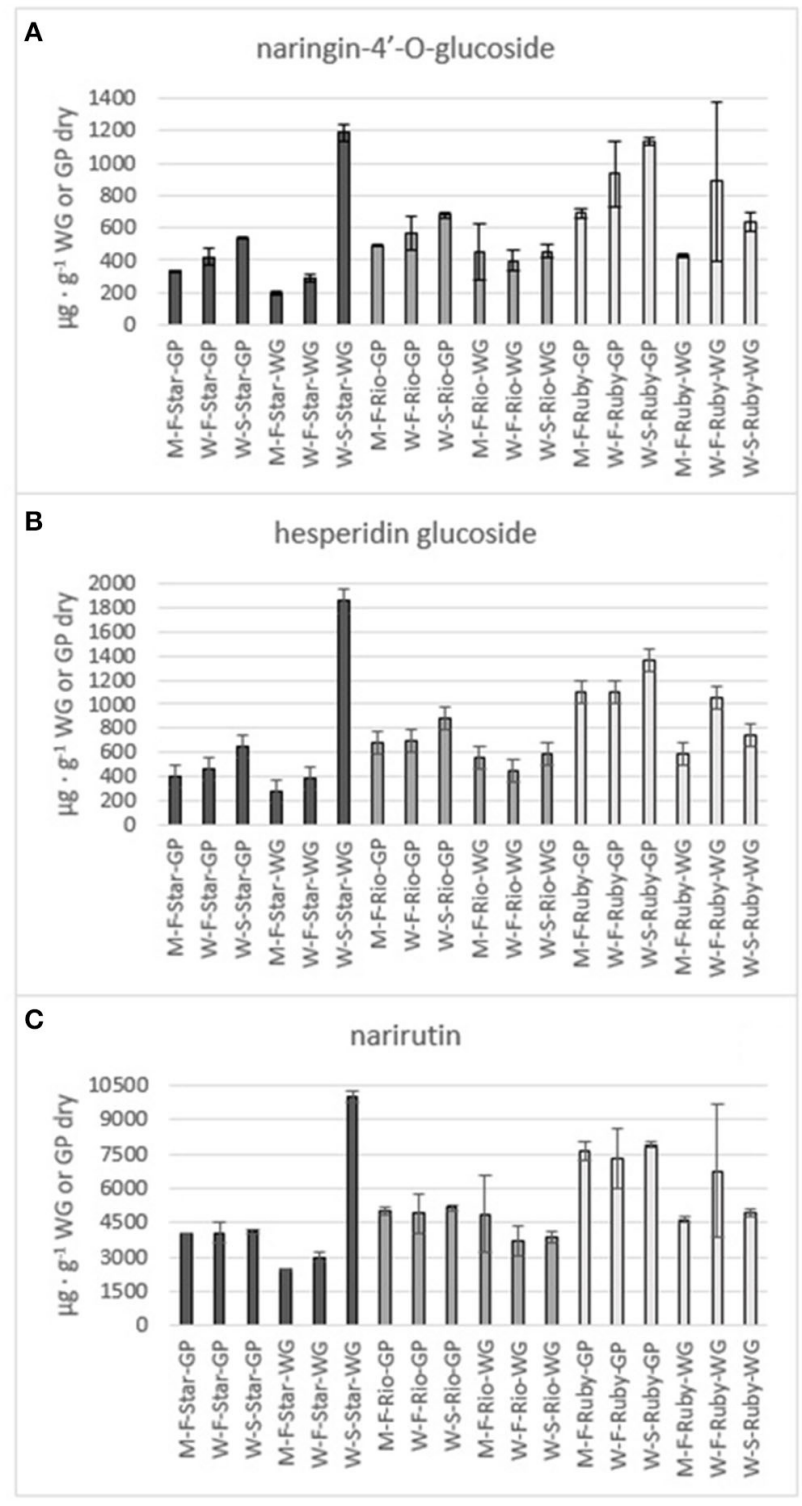
Total amount of the flavones naringin-4'-O- glucoside **(A)**, hesperidin glucoside **(B)** and narirutin **(C)** in the methanol (M) and water (W) extracts of fresh (F) and steam exploded (S) Star Ruby (Star), Rio Red (Rio) and Ruby Red (Ruby) whole grapefruit (WG) and grapefruit juice processing residues (GP). Absence of error bars indicates the Standard Error of the Mean was too small to be visible.

**Figure 7:**
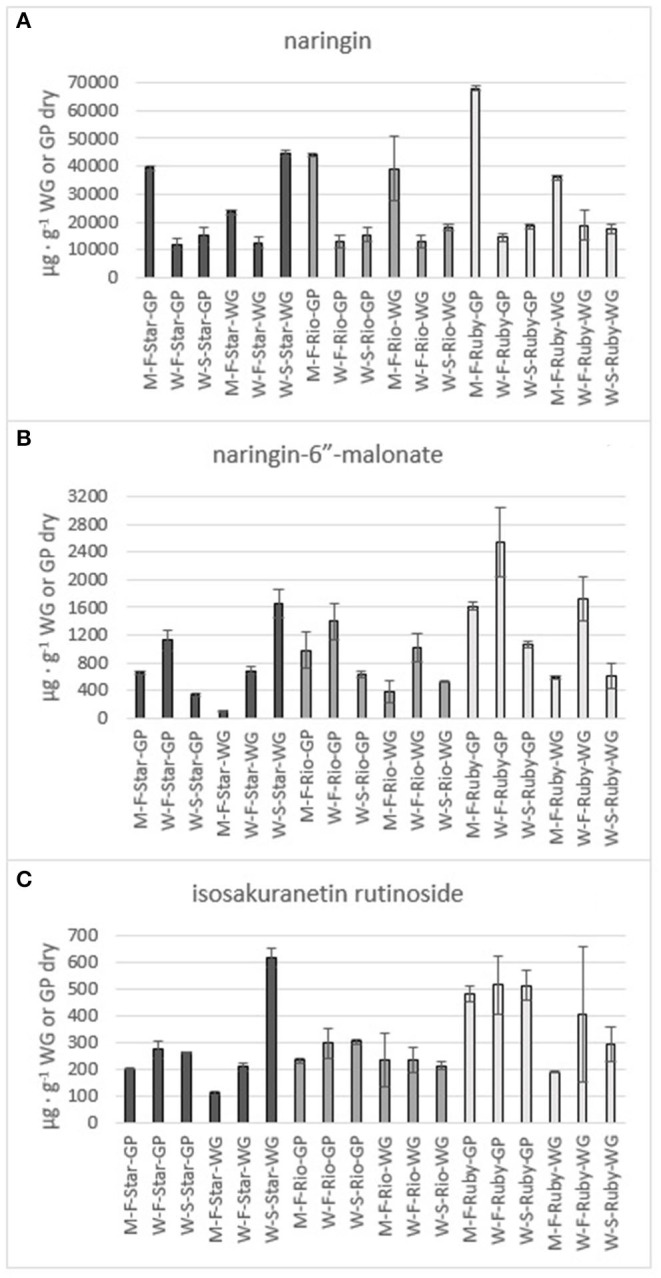
Total amount of the flavones naringin **(A)**, naringin-6” -malonate **(B)** and isosakuranetin rutinoside **(C)** in the methanol (M) and water (W) extracts of fresh (F) and steam exploded (S) Star Ruby (Star), Rio Red (Rio) and Ruby Red (Ruby) whole grapefruit (WG) and grapefruit juice processing residues (GP). Absence of error bars indicates the Standard Error of the Mean was too small to be visible.

**Figure 8:**
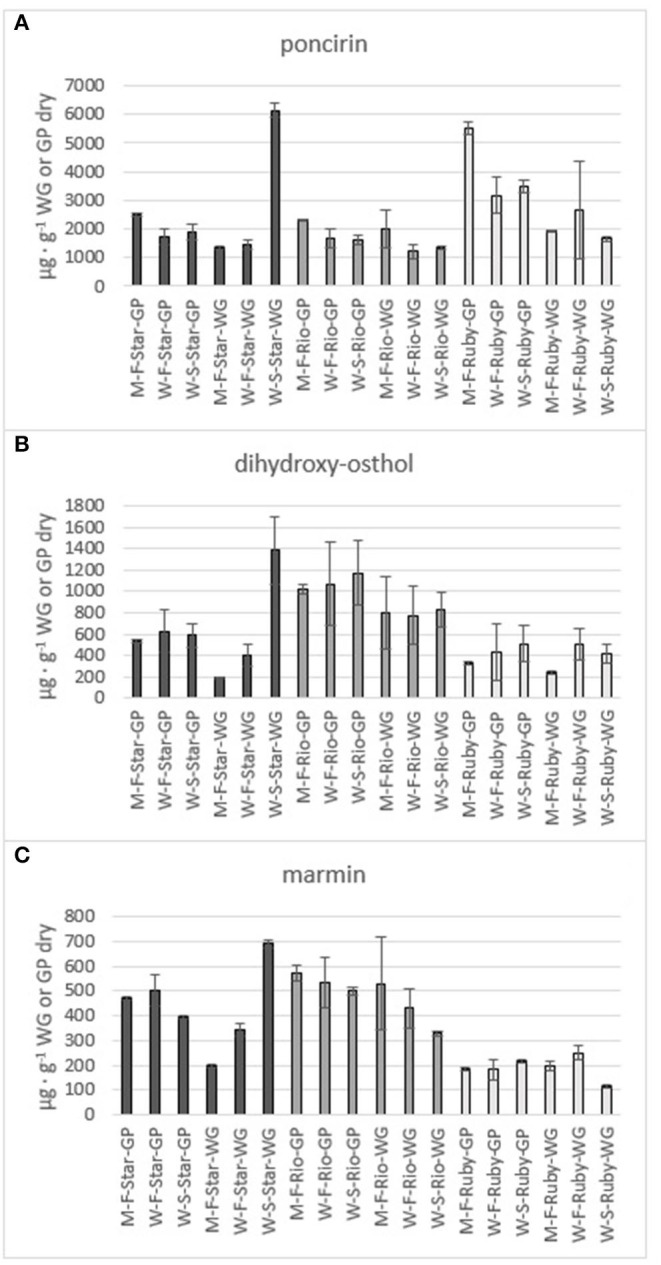
Total amount of the flavone poncirin **(A)** and the coumarins dihydroxy-osthol **(B)** and marmin **(C)** in the methanol (M) and water (W) extracts of fresh (F) and steam exploded (S) Star Ruby (Star), Rio Red (Rio) and Ruby Red (Ruby) whole grapefruit (WG) and grapefruit juice processing residues (GP). Absence of error bars indicates the Standard Error of the Mean was too small to be visible.

The phenolics and flavonoids in methanol extracts of fresh Star Ruby, Rio Red, and Ruby Red WG and GP were compared with the water extracts of fresh and steam-exploded Star Ruby, Rio Red, and Ruby Red WG and GP (Figures 6–8). In 44% of all the extractions performed, water extracts of steam-exploded samples produced the most amount of a given phenolic or flavonoid. For all three varieties, the water extract of steam-exploded GP contained the most amount of naringin-4′-O- glucoside, hesperidin glucoside, and narirutin (Figure 6). There were 250–1,500% more phenolics and flavonoids in the water extract of steam-exploded Star Ruby WG compared with the methanol and or water extracts of fresh Star Ruby WG. In contrast, there were 50–200% more phenolics and flavonoids (excluding naringin) in the fresh Ruby Red WG water extracts compared with the methanol extracts of fresh Ruby Red WG and the water extracts of steam-exploded Ruby Red WG samples. A comprehensive review of the effect of processing conditions on citrus phenolics found that thermal and mechanical processing can lead to a reduction in phenolic content of citrus fruits due to chemical, enzymatic, oxidative, or thermal decomposition, but an increase in phenolic content of processed citrus fruits has also been observed in thermally treated citrus fruits and is thought to be caused by hydrolysis, polymerization, and improved extraction (47).

#### Potential Value of Phenolics and Flavonoids

The citrus bioflavonoid global market is currently valued at approximately 1 billion USD and is expected to grow to 2 billion USD by 2030 (48). The main driver of this growth is the extensively studied health benefits of citrus bioflavonoids (49–53). For example, narirutin has been found to inhibit inflammation (54, 55) and attenuate liver disease (56) and cardiotoxicity (57), and has exhibited antidepressive effects (58). As a result, high-purity narirutin (98%) can sell for as much as 150 USD per kilogram (59). Narirutin was water extracted in amounts of 4,098 μg g^−1^, 5,134 μg g^−1^, and 7,891 μg g^−1^from steam-exploded Star Ruby, Rio Red, and Ruby Red GP, respectively. If we consider the amount of grapefruit that was processed in the 2018–19 citrus season, this would equate to a potential value of 1.1, 8.3, and 8.7 million USD for water-extracted narirutin from steam-exploded Star Ruby, Rio Red, and Ruby Red GP, respectively. Naringin exhibits antioxidative properties (60), enhances bone mass (61), and has been observed to reduce body weight and plasma lipid levels (62). High-purity naringin is valued at 240 USD per kilogram (63). Water extraction of steam-exploded Star Ruby, Rio Red, and Ruby Red GP yielded 15,140-μg g^−1^, 15,334-μg g^−1^, and 18,398-μg g^−1^ naringin, respectively. This would equate to a potential value of 6.6 million (Star Ruby), 40 million (Rio Red), and 33 million (Ruby Red) USD from each of the varieties based on the amount of grapefruit processed during the 2018–19 citrus season. These cumulative values were based on processed red grapefruit from FL and total processed grapefruit from TX and CA, since NASS does not distinguish between red and white grapefruits for those two states (1).

## Conclusions

Steam explosion has the potential to serve as a method for increasing the value of WG and GP from juice processing facilities by extracting valuable compounds, such as pectin, citrus bioflavonoids, sugars, and phenolics rather than converting them to animal feed and molasses. Steam explosion volatilized 56–95% of the juice oil and peel oil in the grapefruit and had similar amounts of *d*-limonene to cold-pressed and hydrodistilled grapefruit peel oils. More pectic hydrocolloids were recovered from GP than from WG for all three varieties and were recovered in a range of 1.138–2.720 g 100 g^−1^. There was an increase in polydispersity and a decrease in viscosity of pectic hydrocolloids from steam exploded GP or WG as compared with acid extracted fresh grapefruit peel. When steam explosion followed by water extraction was compared with methanol or water extraction of fresh GP or WG, steam explosion was capable of recovering the most amount of a given phenolic or flavonoid in 44% of the cases. Naringin was the most abundant compound among the phenolics and flavonoids analyzed in this study and was present in amounts of 12,000–67,000 μg g^−1^. The maximum recovery of the volatiles, pectic hydrocolloids, and narirutin from steam exploded GP has the potential to yield approximately 137 million US dollars of value based on the amount of GP produced in a single season.

## Statement

Mention of trade names or commercial products in this publication is solely for the purpose of providing specific information and does not imply recommendation or endorsement by the USDA-Agricultural Research Service. USDA is an equal opportunity employer.

## Data Availability Statement

The original contributions presented in the study are included in the article/Supplementary Material, further inquiries can be directed to the corresponding author.

## Author Contributions

CD: conceptualization, investigation, methodology, data analysis, writing—original draft, and writing—review and editing. RC: methodology, data analysis, writing—original draft, and writing—review, and editing. JM, JB, and KF: methodology, data analysis, writing—review, and editing. All authors contributed to the article and approved the submitted version.

## Funding

This research was funded by the United States Department of Agriculture as part of the In-House Appropriated Project# 6034-41000-018-00-D.

## Conflict of Interest

The authors declare that the research was conducted in the absence of any commercial or financial relationships that could be construed as a potential conflict of interest.

## Publisher's Note

All claims expressed in this article are solely those of the authors and do not necessarily represent those of their affiliated organizations, or those of the publisher, the editors and the reviewers. Any product that may be evaluated in this article, or claim that may be made by its manufacturer, is not guaranteed or endorsed by the publisher.
