# Supplementary material for: Analysis and Potential Value of Compounds Extracted From Star Ruby, Rio Red, and Ruby Red Grapefruit, and Grapefruit Juice Processing Residues via Steam Explosion
Source: Front Nutr. 2021 Sep 13;8:691663. doi: 10.3389/fnut.2021.691663 (PMC8473638; doi:10.3389/fnut.2021.691663)
Supplement: Supplementary file 1 [file Data_Sheet_1.docx]

Supplementary Material


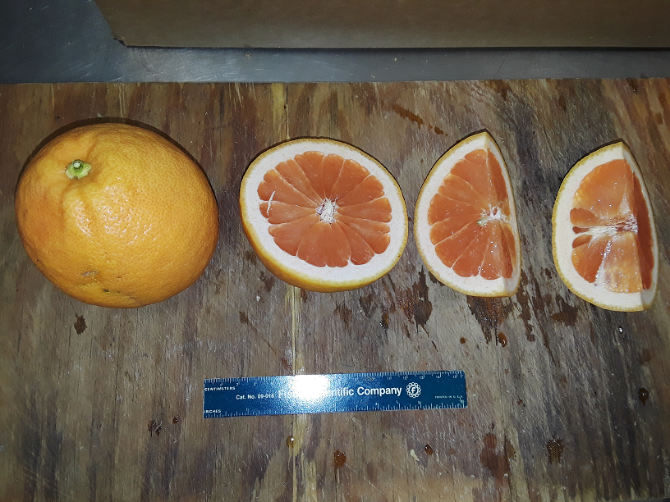


**Figure S1.** Star Ruby Grapefruit


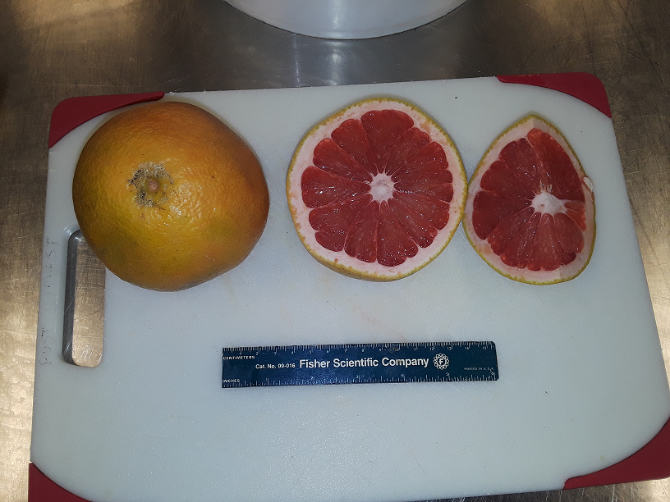


**Figure S2.** Rio Red Grapefruit


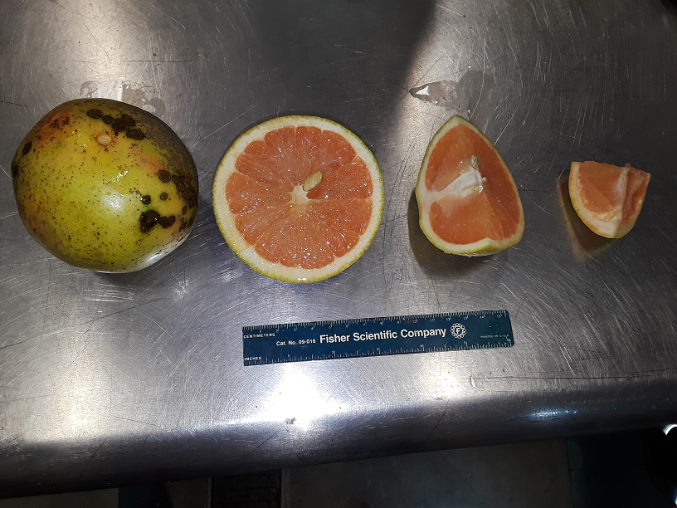


**Figure S3.** Ruby Red Grapefruit


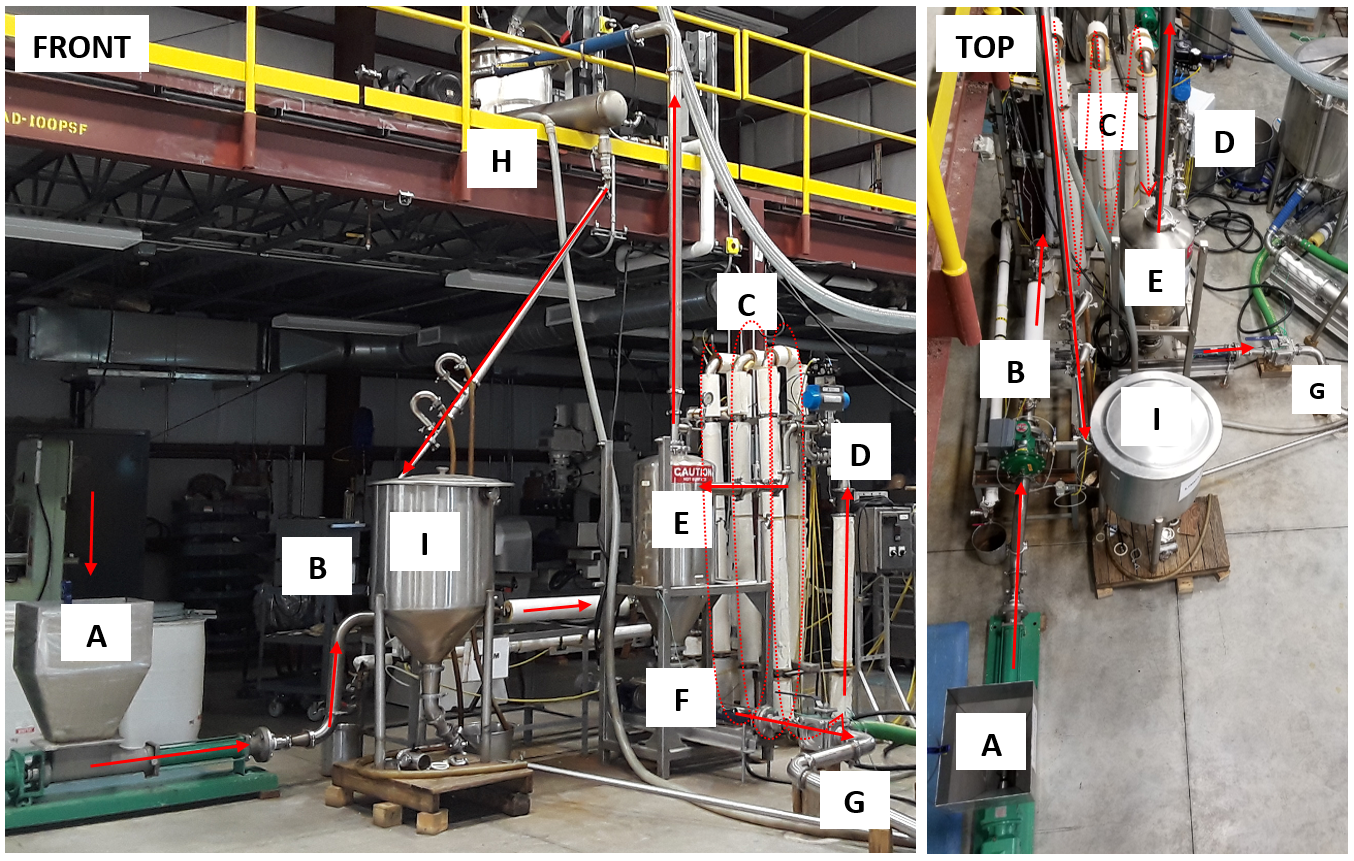


**Figure S4.** Front and top view of continuous pilot scale steam explosion system. Size reduced whole grapefruit (WG) or grapefruit juice processing residues (GP) are fed into a hopper (A) with an auger. The auger conveys WG or GP to a jet cooker (B) that injects steam in to the WG or GP and conveys it to a hold tube (C). The dimensions of the hold tube are such that the WG or GP are exposed to steam from 1-3 minutes until it reaches a pneumatic valve (D) that is set to release pressure at 50 psi. When the set point is reached, the valve opens leading to a drop in pressure that cause the material to explode and propel into the flash tank (E). The solid material is transported via a high solids pump (F) and collected at the end of a stainless steel tube (G). The volatiles travel up a pipe from the flash tank (E) to the condenser (H) and down to a collection tank (I).

**A**

**Figure S5.** HPLC sample chromatogram for compositional sugar analysis of Star Ruby grapefruit juice processing residues.


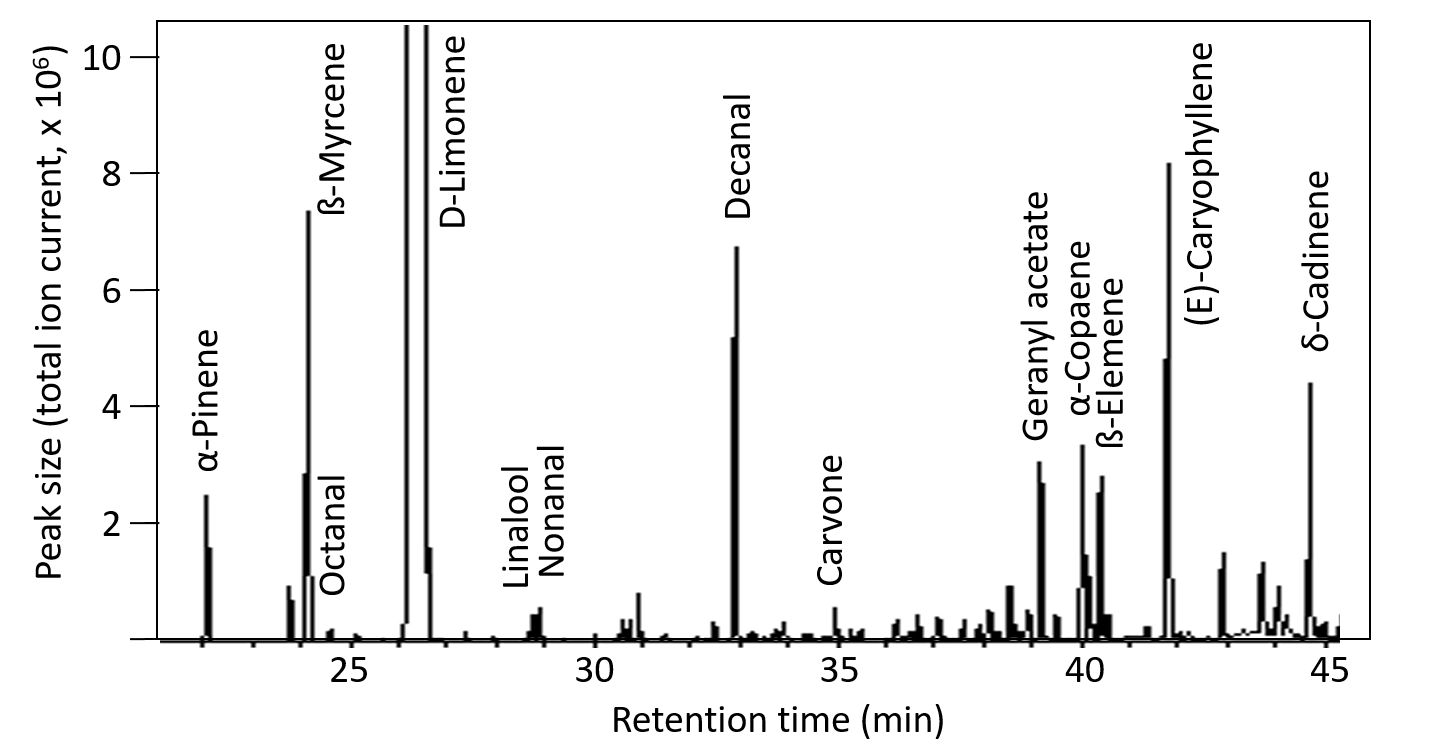


**Figure S6.** GC-MS chromatogram of peel oil extracted from Star Ruby grapefruit.


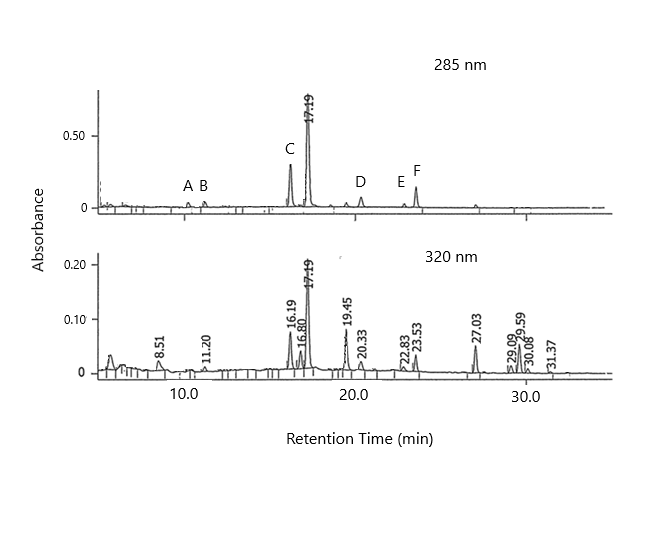


**Figure S7.** HPLC chromatogram of water washed steam exploded grapefruit peel. Peak assignments were made by comparisons of elution times and of ultra violet and mass spectra of known standards. Peak assignments occurred as: A, naringin-4'-O-glucoside; B, hesperidin-O-glucoside; C, narirutin; 17.19 min, naringin; D, naringin-6"-malonate; E, isosakuranetin rutinoside; F, poncirin; 27.03 min, marmin; 29.59 min, nobiletin. The compound eluting at 19.45 min exhibited mass spectra fragmentation consistent with a dihydroxyl osthole unknown.

**Figure S8.** Soluble sugar content of Star Ruby, Rio Red and Ruby Red (A) fresh whole grapefruit (WG), (B) steam exploded (STEX) WG, (C) fresh grapefruit juice processing residues (GP) and (D) STEX GP.

**Figure S9.** Compositional sugar content of Star Ruby, Rio Red and Ruby Red (A) fresh whole grapefruit (WG), (B) steam exploded (STEX) WG, (C) fresh grapefruit juice processing residues (GP) and (D) STEX GP.

**Figure S10.** Degree of methylesterification (% of total GalA residues that are methylesterified at the C6 carboxyl carbon) of recovered pectic hydrocolloids from acid extracted fresh peel, and steam exploded (STEX) grapefruit juice processing peel (GP) and whole grapefruit (WG). Error bars indicate the Standard Error of the Mean. Bars with different lower case letters indicate a statistically significant difference (p > 0.05) within fruit from each variety, bars with different upper case letters indicate a statistically significant difference (p > 0.05) for different varieties for each type of extraction.

| Location | Tulare County, California | Mission, Texas | Florida |
| --- | --- | --- | --- |
| Fruit Type | Packing House | Packing House | Juice Processing |
| Variety | Star Ruby | Rio Red | Ruby Red |
| Date Packed/Processed | May 31, 2018 | Nov 27, 2018 | Feb 1, 2019 (Peel)/Feb 8, 2019 (Whole) |
| Date Acquired | Jul 16, 2018 | Dec 3, 2018 | Feb 1, 2019 (Peel)/Feb 8, 2019 (Whole) |
| Average Whole Fruit Mass (g) | 350 ± 15^a^ | 308 ± 48^b^ | 396 ± 156^c^ |
| Average Peel Mass (g) | 186 ± 13^a^ | 158 ± 29^b^ | 188 ± 89^c^ |
| Average Juice Mass (g) | 163 ± 14^a^ | 135 ± 22^b^ | 197 ± 77^c^ |

**Table S1.** Star Ruby, Rio Red and Ruby Red Grapefruit Information

^a^ Average of six different fruit

^b^ Average of eight different fruit

^c^ Average of ten different fruit

**Table S2.** Star Ruby, Rio Red and Ruby Red Whole Grapefruit (WG) and Grapefruit Juice Processing Residues (GP) Steam Explosion Parameters

| Variety | Star Ruby | Rio Red | Ruby Red |
| --- | --- | --- | --- |
|  |  |  |  |
| Date of Steam Explosion | Jul 20, 2018 | Dec 4, 2018 | Feb 1, 2019 |
| GP Total Mass Run | 82 kg | 153 kg | 137 kg |
| Approximate Total Fruit Used^a^ | 441 | 968 | 729 |
| GP Average Temperature | 140 ± 8 ^o^C | 125 ± 15 ^o^C | 133 ± 13 ^o^C |
| GP Total Run Time | 53 min | 37 min | 38 min |
|  |  |  |  |
| Date of Steam Explosion | Jul 24, 2018 | Dec 6, 2018 | Feb 8, 2019 |
| WG Total Mass Run | 282 kg | 221 kg | 186 kg |
| Approximate Total Fruit Used^b^ | 806 | 718 | 470 |
| WG Average Temperature | 139 ± 1 ^o^C | 136 ± 7 ^o^C | 125 ± 20 ^o^C |
| WG Total Run Time | 59 min | 67 min | 44 min |

^a^Calculated by dividing the total mass run by the average mass of the peel in Table S1

^b^Calculated by dividing the total mass run by the average mass of the fruit in Table S1

**Table S3**. HPLC method time, buffer concentration and flow.

|  | **Buffer** | | |  |
| --- | --- | --- | --- | --- |
| **Time (min)** | **%A** | **%B** | **%C** | **Flow ml/min** |
| 0.00 | 100.00 |  |  | 1.1 |
| 11.00 | 100.00 |  |  | 1.1 |
| 20.00 | 70.00 |  | 30.00 | 1.1 |
| 22.00 | 3.00 | 12.00 | 85.00 | 1.1 |
| 25.50 |  | 100.00 |  | 1.1 |
| 28.50 |  | 100.00 |  | 1.1 |
| 29.00 |  |  | 100.00 | 1.1 |
| 32.00 |  |  | 100.00 | 1.1 |
| 33.00 | 100.00 |  |  | 1.5 |
| 44.00 | 100.00 |  |  | 1.5 |
| 44.50 | 100.00 |  |  | 1.1 |
| 46.00 | 100.00 |  |  | 1.1 |

**Table S4.** Waveform method potentials and pulse times.

| Potential (V) | | Time (millisec) | |
| --- | --- | --- | --- |
| E1 | +0.1 | t1 | 400 |
| E2 | -2.0 | t2 | 20 |
| E3 | +0.6 | t3 | 10 |
| E4 | -0.1 | t4 | 70 |
| E5 | +0.0 | t5 | 0 |
|  |  | ts | 100 |
|  |  | t | 500 |

ts – sampling time

t – total duration of one pulse

|  |  |  |  |  |  | **% based on total ion current** | | | | | | | | |
| --- | --- | --- | --- | --- | --- | --- | --- | --- | --- | --- | --- | --- | --- | --- |
| **Compound** | CAS# | Class 1 | Class 2 | Ret Time | RI | Star-GP-1 | Star-GP-2 | Star-GP-3 | **Average** |  | Star-WG-1 | Star-WG-2 | Star-WG-3 | **Average** |
| **α-Pinene** | 000080-56-8 | Monoterpene | Hydrocarbons | 22.07 | 949 | 0.42 | 0.38 | 0.40 | **0.40** |  | 0.57 | 0.59 | 0.56 | **0.58** |
| **β-Myrcene** | 000123-35-3 | Monoterpene | Hydrocarbons | 24.15 | 997 | 1.46 | 1.30 | 1.39 | **1.38** |  | 1.71 | 1.81 | 1.70 | **1.74** |
| **Octanal** | 000124-13-0 | Aliphatic | Aldehydes | 24.60 | 1008 | 0.04 | 0.03 | 0.04 | **0.04** |  | 0.08 | 0.08 | 0.07 | **0.08** |
| **D-Limonene** | 005989-27-5 | Monoterpene | Hydrocarbons | 26.51 | 1052 | 86.68 | 87.19 | 87.31 | **87.06** |  | 93.68 | 93.51 | 94.00 | **93.73** |
| **Linalool** | 000078-70-6 | Monoterpene | Oxygenated | 28.78 | 1105 | 0.21 | 0.09 | 0.11 | **0.14** |  | 0.15 | 0.15 | 0.06 | **0.12** |
| **Nonanal** | 000124-19-6 | Aliphatic | Aldehydes | 28.89 | 1108 | 0.09 | 0.09 | 0.10 | **0.09** |  | 0.05 | 0.05 | 0.04 | **0.05** |
| **Decanal** | 000112-31-2 | Aliphatic | Aldehydes | 32.92 | 1206 | 1.45 | 1.30 | 1.38 | **1.38** |  | 0.83 | 0.85 | 0.80 | **0.83** |
| **Carvone** | 000099-49-0 | Monoterpene | Oxygenated | 34.96 | 1257 | 0.11 | 0.10 | 0.11 | **0.11** |  | 0.02 | 0.03 | 0.02 | **0.02** |
| **Geranyl acetate** | 000105-87-3 | Monoterpene | Oxygenated | 39.18 | 1371 | 0.50 | 0.45 | 0.48 | **0.48** |  | 0.11 | 0.11 | 0.11 | **0.11** |
| **α-Copaene** | 003856-25-5 | Sesquiterpene | Hydrocarbons | 40.05 | 1396 | 0.68 | 0.61 | 0.65 | **0.65** |  | 0.18 | 0.19 | 0.18 | **0.18** |
| **β-Elemene** | 000515-13-9 | Sesquiterpene | Hydrocarbons | 40.41 | 1406 | 0.52 | 0.47 | 0.50 | **0.50** |  | 0.10 | 0.10 | 0.10 | **0.10** |
| **(E)-Caryophyllene** | 000087-44-5 | Sesquiterpene | Hydrocarbons | 41.80 | 1448 | 1.59 | 1.44 | 1.53 | **1.52** |  | 0.56 | 0.58 | 0.56 | **0.57** |
| **δ-Cadinene** | 000483-76-1 | Sesquiterpene | Hydrocarbons | 44.71 | 1539 | 0.78 | 0.71 | 0.76 | **0.75** |  | 0.20 | 0.22 | 0.18 | **0.20** |

**Table S5.** Peak area percent based on total ion current for each replicate and their average of compounds found in the condensed volatiles from the steam explosion of Star Ruby (Star) whole grapefruit (WG) and grapefruit juice processing residues (GP).

**Table S6**. Peak area percent based on total ion current for each replicate and their average of compounds found in the condensed volatiles from the steam explosion of Ruby Red (Ruby) whole grapefruit (WG) and grapefruit juice processing residues (GP).

|  |  |  |  |  |  | **% based on total ion current** | | | | | | | | | | |
| --- | --- | --- | --- | --- | --- | --- | --- | --- | --- | --- | --- | --- | --- | --- | --- | --- |
| **Compound** | CAS# | Class 1 | Class 2 | Ret Time | RI | Ruby-GP-1 | Ruby-GP-2 | Ruby-GP-3 | Ruby-GP-4 | **Average** |  | Ruby-WG-1 | Ruby-WG-2 | Ruby-WG-3 | Ruby-WG-4 | **Average** |
| **α-Pinene** | 000080-56-8 | Monoterpene | Hydrocarbons | 21.66 | 949 | 0.39 | 0.35 | 0.34 | 0.36 | **0.36** |  | 0.49 | 0.48 | 0.51 | 0.50 | **0.49** |
| **β-Myrcene** | 000123-35-3 | Monoterpene | Hydrocarbons | 23.789 | 998 | 1.93 | 1.72 | 1.45 | 1.53 | **1.66** |  | 1.78 | 1.77 | 1.65 | 1.60 | **1.70** |
| **Octanal** | 000124-13-0 | Aliphatic | Aldehydes | 24.199 | 1007 | 0.08 | 0.07 | 0.06 | 0.07 | **0.07** |  | 0.22 | 0.22 | 0.20 | 0.19 | **0.21** |
| **D-Limonene** | 005989-27-5 | Monoterpene | Hydrocarbons | 26.104 | 1052 | 87.83 | 89.17 | 87.71 | 87.07 | **87.94** |  | 92.18 | 92.26 | 90.77 | 90.51 | **91.43** |
| **Linalool** | 000078-70-6 | Monoterpene | Oxygenated | 28.352 | 1105 | 0.16 | 0.14 | 0.17 | 0.18 | **0.16** |  | 0.12 | 0.12 | 0.25 | 0.25 | **0.18** |
| **Nonanal** | 000124-19-6 | Aliphatic | Aldehydes | 28.469 | 1108 | 0.07 | 0.05 | 0.07 | 0.08 | **0.07** |  | 0.06 | 0.06 | 0.00 | 0.00 | **0.03** |
| **Decanal** | 000112-31-2 | Aliphatic | Aldehydes | 32.716 | 1214 | 0.05 | 0.05 | 0.09 | 0.10 | **0.07** |  | 0.02 | 0.02 | 0.06 | 0.06 | **0.04** |
| **Carvone** | 000099-49-0 | Monoterpene | Oxygenated | 34.516 | 1261 | 1.64 | 1.49 | 1.59 | 1.67 | **1.60** |  | 0.47 | 0.46 | 0.56 | 0.61 | **0.52** |
| **Geranyl acetate** | 000105-87-3 | Monoterpene | Oxygenated | 38.751 | 1378 | 0.13 | 0.12 | 0.12 | 0.13 | **0.12** |  | 0.08 | 0.08 | 0.11 | 0.12 | **0.10** |
| **α-Copaene** | 003856-25-5 | Sesquiterpene | Hydrocarbons | 39.591 | 1402 | 0.35 | 0.32 | 0.30 | 0.31 | **0.32** |  | 0.22 | 0.21 | 0.22 | 0.22 | **0.22** |
| **β-Elemene** | 000515-13-9 | Sesquiterpene | Hydrocarbons | 39.942 | 1413 | 0.31 | 0.28 | 0.25 | 0.26 | **0.28** |  | 0.13 | 0.13 | 0.12 | 0.12 | **0.12** |
| **(E)-Caryophyllene** | 000087-44-5 | Sesquiterpene | Hydrocarbons | 41.357 | 1455 | 1.36 | 1.22 | 0.98 | 0.00 | **0.89** |  | 0.78 | 0.77 | 0.66 | 0.64 | **0.71** |
| **δ-Cadinene** | 000483-76-1 | Sesquiterpene | Hydrocarbons | 44.25 | 1547 | 0.34 | 0.30 | 0.22 | 0.24 | **0.27** |  | 0.24 | 0.24 | 0.20 | 0.20 | **0.22** |

|  |  |  |  |  |  | **% based on total ion current** | | | | | | | | | | |
| --- | --- | --- | --- | --- | --- | --- | --- | --- | --- | --- | --- | --- | --- | --- | --- | --- |
| **Compound** | CAS# | Class 1 | Class 2 | Ret Time | RI | Rio-GP-1 | Rio-GP-2 | Rio-GP-3 | Rio-GP-4 | **Average** |  | TX-WG-1 | TX-WG-2 | TX-WG-3 | TX-WG-4 | **Average** |
| **α-Pinene** | 000080-56-8 | Monoterpene | Hydrocarbons | 21.66 | 949 | 0.72 | 0.73 | 0.68 | 0.74 | **0.72** |  | 0.63 | 0.59 | 0.55 | 0.56 | **0.58** |
| **β-Myrcene** | 000123-35-3 | Monoterpene | Hydrocarbons | 23.789 | 998 | 2.52 | 2.56 | 2.21 | 2.32 | **2.40** |  | 2.42 | 2.24 | 2.00 | 2.03 | **2.17** |
| **Octanal** | 000124-13-0 | Aliphatic | Aldehydes | 24.199 | 1007 | 0.24 | 0.24 | 0.21 | 0.21 | **0.22** |  | 0.27 | 0.26 | 0.21 | 0.22 | **0.24** |
| **D-Limonene** | 005989-27-5 | Monoterpene | Hydrocarbons | 26.104 | 1052 | 91.99 | 91.95 | 90.53 | 90.48 | **91.24** |  | 91.84 | 91.11 | 91.09 | 91.18 | **91.31** |
| **Linalool** | 000078-70-6 | Monoterpene | Oxygenated | 28.352 | 1105 | 0.15 | 0.15 | 0.25 | 0.26 | **0.20** |  | 0.16 | 0.15 | 0.09 | 0.28 | **0.17** |
| **Nonanal** | 000124-19-6 | Aliphatic | Aldehydes | 28.469 | 1108 | 0.07 | 0.07 | 0.00 | 0.00 | **0.03** |  | 0.08 | 0.08 | 0.00 | 0.00 | **0.04** |
| **Decanal** | 000112-31-2 | Aliphatic | Aldehydes | 32.716 | 1214 | 0.02 | 0.02 | 0.08 | 0.50 | **0.16** |  | 0.00 | 0.00 | 0.08 | 0.05 | **0.03** |
| **Carvone** | 000099-49-0 | Monoterpene | Oxygenated | 34.516 | 1261 | 0.17 | 0.18 | 0.32 | 0.03 | **0.17** |  | 0.13 | 0.13 | 0.02 | 0.24 | **0.13** |
| **Geranyl acetate** | 000105-87-3 | Monoterpene | Oxygenated | 38.751 | 1378 | 0.04 | 0.05 | 0.07 | 0.39 | **0.14** |  | 0.06 | 0.05 | 0.16 | 0.07 | **0.08** |
| **α-Copaene** | 003856-25-5 | Sesquiterpene | Hydrocarbons | 39.591 | 1402 | 0.16 | 0.16 | 0.14 | 0.21 | **0.17** |  | 0.21 | 0.20 | 0.04 | 0.18 | **0.15** |
| **β-Elemene** | 000515-13-9 | Sesquiterpene | Hydrocarbons | 39.942 | 1413 | 0.16 | 0.16 | 0.14 | 0.03 | **0.12** |  | 0.17 | 0.17 | 0.02 | 0.14 | **0.12** |
| **(E)-Caryophyllene** | 000087-44-5 | Sesquiterpene | Hydrocarbons | 41.357 | 1455 | 0.51 | 0.52 | 0.38 | 0.02 | **0.36** |  | 0.64 | 0.61 | 0.07 | 0.54 | **0.46** |
| **δ-Cadinene** | 000483-76-1 | Sesquiterpene | Hydrocarbons | 44.25 | 1547 | 0.16 | 0.16 | 0.12 | 0.05 | **0.12** |  | 0.24 | 0.23 | 0.04 | 0.20 | **0.18** |

**Table S7**. Peak area percent based on total ion current for each replicate and their average of compounds found in the condensed volatiles from the steam explosion of Rio Red (Rio) whole grapefruit (WG) and grapefruit juice processing residues (GP).

|  |  |  |  |  |  | **% based on total ion current** | | | |
| --- | --- | --- | --- | --- | --- | --- | --- | --- | --- |
| **Compound** | CAS# | Class 1 | Class 2 | Ret Time | RI | Rio-CPC-GP-1 | Rio-CPC-GP-2 | Rio-CPC-GP--3 | **Average** |
| **α-Pinene** | 000080-56-8 | Monoterpene | Hydrocarbons | 21.66 | 949 | 0.66 | 0.57 | 0.61 | **0.62** |
| **β-Myrcene** | 000123-35-3 | Monoterpene | Hydrocarbons | 23.79 | 998 | 1.84 | 1.77 | 1.76 | **1.79** |
| **Octanal** | 000124-13-0 | Aliphatic | Aldehydes | 24.20 | 1007 | 0.31 | 0.26 | 0.29 | **0.28** |
| **D-Limonene** | 005989-27-5 | Monoterpene | Hydrocarbons | 26.10 | 1052 | 93.40 | 92.98 | 93.12 | **93.17** |
| **Linalool** | 000078-70-6 | Monoterpene | Oxygenated | 28.35 | 1105 | 0.10 | 0.09 | 0.07 | **0.09** |
| **Nonanal** | 000124-19-6 | Aliphatic | Aldehydes | 28.47 | 1108 | 0.09 | 0.08 | 0.07 | **0.08** |
| **Decanal** | 000112-31-2 | Aliphatic | Aldehydes | nd | nd | nd | nd | nd | **nd** |
| **Carvone** | 000099-49-0 | Monoterpene | Oxygenated | nd | nd | nd | nd | nd | **nd** |
| **Geranyl acetate** | 000105-87-3 | Monoterpene | Oxygenated | 38.75 | 1378 | 0.05 | 0.04 | 0.04 | **0.04** |
| **α-Copaene** | 003856-25-5 | Sesquiterpene | Hydrocarbons | 39.59 | 1402 | 0.18 | 0.15 | 0.14 | **0.16** |
| **β-Elemene** | 000515-13-9 | Sesquiterpene | Hydrocarbons | 39.94 | 1413 | 0.18 | 0.15 | 0.14 | **0.16** |
| **(E)-Caryophyllene** | 000087-44-5 | Sesquiterpene | Hydrocarbons | 41.36 | 1455 | 0.48 | 0.39 | 0.33 | **0.40** |
| **δ-Cadinene** | 000483-76-1 | Sesquiterpene | Hydrocarbons | 44.25 | 1547 | 0.21 | 0.16 | 0.16 | **0.18** |

**Table S8**. Peak area percent based on total ion current for each replicate and their average of compounds found in the cold pressed and centrifuged (CPC) peel oil of Rio Red (Rio) grapefruit juice processing residues (GP).

**Table S9**. First replicate of total amount of naringin-4’-O-glucoside, hesperidin glucoside, narirutin, naringin, naringin-6”-malonate, isosakuranetin rutinoside, poncirin, dihydroxy-osthol and marmin in the methanol and water extracts of fresh and steam exploded Star Ruby, Rio Red and Ruby Red whole grapefruit (WG) and grapefruit juice processing residues (GP) in µg g^-1^ WG or GP dry.

| Rep 1 |  | Star Ruby | | | | | | Rio Red | | | | | | Ruby Red | | | | | |
| --- | --- | --- | --- | --- | --- | --- | --- | --- | --- | --- | --- | --- | --- | --- | --- | --- | --- | --- | --- |
|  |  | GP | | | WG | | | GP | | | WG | | | GP | | | WG | | |
|  |  | MeOH | Water | | MeoH | Water | | MeOH | Water | | MeOH | Water | | MeOH | Water | | MeOH | Water | |
| compound | ET | Fresh | Fresh | Steam | Fresh | Fresh | Steam | Fresh | Fresh | Steam | Fresh | Fresh | Steam | Fresh | Fresh | Steam | Fresh | Fresh | Steam |
| naringin-4’-O-glucoside | 10.4 | 327 | 460 | 532 | 191 | 299 | 1212 | 480 | 662 | 691 | 352 | 462 | 478 | 689 | 1105 | 1157 | 437 | 600 | 615 |
| hesperidin glucoside | 11.4 | 397 | 516 | 682 | 275 | 422 | 1889 | 682 | 799 | 909 | 443 | 507 | 631 | 1077 | 1284 | 1404 | 601 | 698 | 758 |
| narirutin | 16.4 | 3988 | 4434 | 4171 | 2421 | 3125 | 10279 | 5144 | 5653 | 5296 | 3898 | 4308 | 3909 | 7457 | 8358 | 8032 | 4749 | 5350 | 4859 |
| naringin | 17.4 | 38555 | 14212 | 18476 | 22962 | 14233 | 45398 | 44525 | 15101 | 17989 | 32474 | 14649 | 19318 | 67325 | 15701 | 19499 | 36920 | 17585 | 19503 |
| naringin-6”-malonate | 20.5 | 658 | 1259 | 366 | 101 | 742 | 1811 | 860 | 1650 | 682 | 307 | 1175 | 517 | 1566 | 3020 | 1107 | 611 | 2037 | 759 |
| isosakuranetin rutinoside | 23.0 | 200 | 302 | 269 | 110 | 222 | 645 | 232 | 346 | 308 | 182 | 277 | 231 | 468 | 604 | 555 | 193 | 289 | 268 |
| poncirin | 23.6 | 2473 | 2002 | 2175 | 1313 | 1572 | 6399 | 2333 | 1961 | 1764 | 1616 | 1426 | 1433 | 5445 | 3688 | 3690 | 1970 | 1839 | 1759 |
| dihydroxy-osthol | 19.7 | 524 | 850 | 720 | 182 | 515 | 1749 | 982 | 1499 | 1515 | 600 | 1076 | 1020 | 318 | 727 | 688 | 244 | 540 | 523 |
| marmin | 27.2 | 468 | 553 | 399 | 193 | 361 | 709 | 547 | 598 | 519 | 414 | 494 | 323 | 182 | 216 | 221 | 207 | 252 | 119 |

**Table S10**. Second replicate of total amount of naringin-4’-O-glucoside, hesperidin glucoside, narirutin, naringin, naringin-6”-malonate, isosakuranetin rutinoside, poncirin, dihydroxy-osthol and marmin in the methanol and water extracts of fresh and steam exploded Star Ruby, Rio Red and Ruby Red whole grapefruit (WG) and grapefruit juice processing residues (GP) in µg g^-1^ WG or GP dry.

| Rep 2 |  | Star Ruby | | | | | | Rio Red | | | | | | Ruby Red | | | | | |
| --- | --- | --- | --- | --- | --- | --- | --- | --- | --- | --- | --- | --- | --- | --- | --- | --- | --- | --- | --- |
|  |  | GP | | | WG | | | GP | | | WG | | | GP | | | WG | | |
|  |  | MeOH | Water | | MeOH | Water | | MeOH | Water | | MeOH | Water | | MeOH | Water | | MeOH | Water | |
|  | ET | Fresh | Fresh | Steam | Fresh | Fresh | Steam | Fresh | Fresh | Steam | Fresh | Fresh | Steam | Fresh | Fresh | Steam | Fresh | Fresh | Steam |
| naringin-4’-O-glucoside | 10.4 | 329 | 434 | 541 | 186 | 298 | 1132 | 490 | 585 | 663 | 355 | 405 | 407 | 715 | 987 | 1109 | 421 | 1456 | 595 |
| hesperidin glucoside | 11.4 | 398 | 478 | 651 | 278 | 389 | 1781 | 672 | 742 | 878 | 427 | 476 | 562 | 1121 | 1169 | 1367 | 601 | 1767 | 724 |
| narirutin | 16.4 | 4057 | 4197 | 4101 | 2437 | 3085 | 9750 | 4850 | 5109 | 5066 | 3898 | 3734 | 3631 | 8103 | 7754 | 7776 | 4612 | 10109 | 4781 |
| naringin | 17.4 | 40226 | 11666 | 14568 | 23149 | 12916 | 43066 | 43575 | 13655 | 15248 | 32790 | 13409 | 17634 | 68855 | 14647 | 18215 | 35917 | 24542 | 16831 |
| naringin-6”-malonate | 20.5 | 625 | 1146 | 339 | 96 | 703 | 1731 | 795 | 1419 | 623 | 287 | 1097 | 529 | 1670 | 2583 | 1061 | 599 | 1393 | 672 |
| isosakuranetin rutinoside | 23.0 | 202 | 285 | 261 | 110 | 220 | 576 | 226 | 317 | 312 | 180 | 246 | 206 | 515 | 552 | 538 | 190 | 698 | 252 |
| poncirin | 23.6 | 2557 | 1760 | 1807 | 1326 | 1492 | 5929 | 2302 | 1749 | 1652 | 1615 | 1240 | 1293 | 5775 | 3383 | 3512 | 1922 | 4644 | 1616 |
| dihydroxy-osthol | 20 | 531 | 576 | 522 | 186 | 358 | 1231 | 1001 | 963 | 975 | 598 | 714 | 739 | 340 | 342 | 483 | 242 | 627 | 368 |
| marmin | 27 | 466 | 518 | 393 | 197 | 356 | 678 | 563 | 591 | 495 | 429 | 455 | 325 | 192 | 194 | 212 | 214 | 278 | 111 |

**Table S11**. Third replicate of total amount of naringin-4’-O-glucoside, hesperidin glucoside, narirutin, naringin, naringin-6”-malonate, isosakuranetin rutinoside, poncirin, dihydroxy-osthol and marmin in the methanol and water extracts of fresh and steam exploded Star Ruby, Rio Red and Ruby Red whole grapefruit (WG) and grapefruit juice processing residues (GP) in µg g^-1^ WG or GP dry.

| Rep 3 |  | Star Ruby | | | | | | Rio Red | | | | | | Ruby Red | | | | | |
| --- | --- | --- | --- | --- | --- | --- | --- | --- | --- | --- | --- | --- | --- | --- | --- | --- | --- | --- | --- |
|  |  | Grapefruit Processing Waste | | | Whole Grapefruit | | | Grapefruit Processing Waste | | | Whole Grapefruit | | | Grapefruit Processing Waste | | | Whole Grapefruit | | |
|  |  | MeOH | Water | | MeOH | Water | | MeOH | Water | | MeOH | Water | | MeOH | Water | | MeOH | Water | |
| Compound | ET | Fresh | Fresh | Steam | Fresh | Fresh | Steam | Fresh | Fresh | Steam | Fresh | Fresh | Steam | Fresh | Fresh | Steam | Fresh | Fresh | Steam |
| naringin-4’-O-glucoside | 10.4 | 341 | 365 | 529 | 205 | 265 | 1216 | 496 | 458 | 677 | 656 | 329 | 475 | 664 | 713 | 1141 | 416 | 606 | 700 |
| hesperidin glucoside | 11.4 | 402 | 403 | 622 | 283 | 336 | 1893 | 685 | 544 | 850 | 807 | 359 | 581 | 1115 | 860 | 1312 | 576 | 681 | 756 |
| narirutin | 16.4 | 4032 | 3559 | 4024 | 2510 | 2768 | 10060 | 5104 | 3978 | 5042 | 6789 | 3034 | 4041 | 7382 | 5867 | 7866 | 4516 | 4850 | 5134 |
| naringin | 17.4 | 39314 | 10080 | 12376 | 24228 | 10771 | 45045 | 44281 | 10673 | 12765 | 52304 | 10266 | 17075 | 67335 | 13018 | 17481 | 34907 | 14110 | 16560 |
| naringin-6”-malonate | 20.5 | 660 | 959 | 313 | 114 | 617 | 1419 | 1278 | 1121 | 590 | 562 | 788 | 522 | 1611 | 2027 | 1014 | 562 | 1759 | 399 |
| isosakuranetin rutinoside | 23.0 | 207 | 239 | 259 | 118 | 194 | 635 | 246 | 237 | 295 | 351 | 189 | 207 | 462 | 392 | 454 | 188 | 233 | 371 |
| poncirin | 23.6 | 2507 | 1461 | 1654 | 1382 | 1286 | 6122 | 2312 | 1316 | 1445 | 2790 | 955 | 1318 | 5387 | 2473 | 3232 | 1878 | 1513 | 1632 |
| dihydroxy-osthol | 20 | 543 | 462 | 521 | 198 | 324 | 1178 | 1067 | 749 | 1026 | 1194 | 541 | 723 | 318 | 224 | 358 | 221 | 345 | 371 |
| marmin | 27 | 476 | 436 | 386 | 203 | 318 | 688 | 606 | 415 | 485 | 747 | 339 | 338 | 181 | 138 | 212 | 177 | 220 | 116 |

**Table S12**. Averages calculated from triplicate analysis of total amount of naringin-4’-O-glucoside, hesperidin glucoside, narirutin, naringin, naringin-6”-malonate, isosakuranetin rutinoside, poncirin, dihydroxy-osthol and marmin in the methanol and water extracts of fresh and steam exploded Star Ruby, Rio Red and Ruby Red whole grapefruit (WG) and grapefruit juice processing residues (GP) in µg g^-1^ WG or GP dry.

| Average |  | Star Ruby | | | | | | Rio Red | | | | | | Ruby Red | | | | | |
| --- | --- | --- | --- | --- | --- | --- | --- | --- | --- | --- | --- | --- | --- | --- | --- | --- | --- | --- | --- |
|  |  | GP | | | WG | | | GP | | | WG | | | GP | | | WG | | |
|  |  | MeOH | Water | | MeOH | Water | | MeOH | Water | | MeOH | Water | | MeOH | Water | | MeOH | Water | |
| Compound | ET | Fresh | Fresh | Steam | Fresh | Fresh | Steam | Fresh | Fresh | Steam | Fresh | Fresh | Steam | Fresh | Fresh | Steam | Fresh | Fresh | Steam |
| naringin-4’-O-glucoside | 10.4 | 332 | 419 | 534 | 194 | 287 | 1187 | 488 | 569 | 677 | 454 | 399 | 453 | 689 | 935 | 1135 | 425 | 887 | 637 |
| hesperidin glucoside | 11.4 | 399 | 466 | 652 | 279 | 382 | 1854 | 679 | 695 | 879 | 559 | 447 | 592 | 1104 | 1104 | 1361 | 592 | 1049 | 746 |
| narirutin | 16.4 | 4026 | 4064 | 4099 | 2456 | 2993 | 10030 | 5033 | 4913 | 5135 | 4862 | 3692 | 3860 | 7648 | 7327 | 7891 | 4626 | 6770 | 4925 |
| naringin | 17.4 | 39365 | 11986 | 15140 | 23446 | 12640 | 44503 | 44127 | 13143 | 15334 | 39189 | 12774 | 18009 | 67838 | 14455 | 18398 | 35915 | 18746 | 17631 |
| naringin-6”-malonate | 20.5 | 647 | 1122 | 339 | 104 | 687 | 1654 | 977 | 1397 | 632 | 385 | 1020 | 522 | 1616 | 2543 | 1061 | 591 | 1730 | 610 |
| isosakuranetin rutinoside | 23.0 | 203 | 276 | 263 | 113 | 212 | 618 | 235 | 300 | 305 | 238 | 237 | 215 | 482 | 516 | 515 | 190 | 407 | 297 |
| poncirin | 23.6 | 2512 | 1741 | 1879 | 1340 | 1450 | 6150 | 2315 | 1675 | 1620 | 2007 | 1207 | 1348 | 5536 | 3181 | 3478 | 1923 | 2665 | 1669 |
| dihydroxy-osthol | 19.7 | 532 | 630 | 588 | 189 | 399 | 1386 | 1017 | 1071 | 1172 | 797 | 777 | 827 | 325 | 431 | 510 | 236 | 504 | 421 |
| marmin | 27.2 | 470 | 502 | 392 | 197 | 345 | 692 | 572 | 535 | 499 | 530 | 429 | 329 | 185 | 183 | 215 | 199 | 250 | 115 |

**Table S13**. Standard deviations calculated from triplicate analysis of total amount of naringin-4’-O-glucoside, hesperidin glucoside, narirutin, naringin, naringin-6”-malonate, isosakuranetin rutinoside, poncirin, dihydroxy-osthol and marmin in the methanol and water extracts of fresh and steam exploded Star Ruby, Rio Red and Ruby Red whole grapefruit (WG) and grapefruit juice processing residues (GP) in µg g^-1^ WG or GP dry.

| STD DEV |  | Star Ruby | | | | | | Rio Red | | | | | | Ruby Red | | | | | |
| --- | --- | --- | --- | --- | --- | --- | --- | --- | --- | --- | --- | --- | --- | --- | --- | --- | --- | --- | --- |
|  |  | GP | | | WG | | | GP | | | WG | | | GP | | | WG | | |
|  |  | MeOH | Water | | MeOH | Water | | MeOH | Water | | MeOH | Water | | MeOH | Water | | MeOH | Water | |
|  |  | Fresh | Fresh | Steam | Fresh | Fresh | Steam | Fresh | Fresh | Steam | Fresh | Fresh | Steam | Fresh | Fresh | Steam | Fresh | Fresh | Steam |
| naringin-4’-O-glucoside | 10.4 | 7 | 49 | 6 | 9 | 20 | 47 | 8 | 103 | 14 | 175 | 67 | 40 | 25 | 201 | 24 | 11 | 492 | 55 |
| hesperidin glucoside | 11.4 | 3 | 57 | 30 | 4 | 43 | 63 | 7 | 134 | 29 | 215 | 78 | 36 | 24 | 219 | 46 | 15 | 622 | 19 |
| narirutin | 16.4 | 35 | 452 | 73 | 47 | 195 | 266 | 160 | 855 | 140 | 1669 | 638 | 209 | 396 | 1299 | 130 | 117 | 2903 | 186 |
| naringin | 17.4 | 837 | 2084 | 3090 | 683 | 1747 | 1257 | 493 | 2258 | 2613 | 11359 | 2259 | 1167 | 880 | 1352 | 1022 | 1006 | 5312 | 1627 |
| naringin-6”-malonate | 20.5 | 20 | 152 | 26 | 9 | 64 | 207 | 262 | 266 | 46 | 153 | 204 | 6 | 52 | 498 | 46 | 25 | 323 | 188 |
| isosakuranetin rutinoside | 23.0 | 4 | 33 | 5 | 4 | 16 | 37 | 10 | 56 | 9 | 98 | 44 | 14 | 29 | 110 | 54 | 3 | 254 | 65 |
| poncirin | 23.6 | 42 | 271 | 268 | 37 | 148 | 236 | 16 | 329 | 162 | 678 | 237 | 74 | 210 | 632 | 231 | 46 | 1722 | 79 |
| dihydroxy-osthol | 19.7 | 10 | 199 | 114 | 8 | 102 | 315 | 45 | 386 | 298 | 343 | 273 | 167 | 13 | 263 | 167 | 13 | 144 | 89 |
| marmin | 27.2 | 5 | 60 | 6 | 5 | 24 | 16 | 31 | 104 | 17 | 188 | 81 | 8 | 6 | 40 | 5 | 20 | 29 | 4 |
